# Supplementary material for: Anti-CD19 CARs displayed at the surface of lentiviral vector particles promote transduction of target-expressing cells
Source: Mol Ther Methods Clin Dev. 2021 Feb 24;21:42–53. doi: 10.1016/j.omtm.2021.02.013 (PMC7966970; doi:10.1016/j.omtm.2021.02.013)
Supplement: Document S1. Supplemental materials and methods, Figures S1–S10, and Table S1 [file mmc1.pdf]

OMTM, Volume *21*

Supplemental information

Anti-CD19 CARs displayed at the surface of lentiviral vector particles promote transduction of target-expressing cells

Nicole Cordes, Carolin Kolbe, Dominik Lock, Tatjana Holzer, Deborah Althoff, Daniel Schäfer, Franziska Blaeschke, Bettina Kotter, Sandra Karitzky, Claudia Rossig, Toni Cathomen, Tobias Feuchtinger, Iris Bürger, Mario Assenmacher, Thomas Schaser, and Andrew D. Kaiser

Supplemental Methods

Monoculture transduction

One day prior transduction 200 µl of B cells with a density of 1 x10^6 cells/ml were seeded in 200 µl StemMACS HSC Expansion Medium XF supplemented with 50 IU/ml IL-4, CD40-L with 3.6 µl/ml cross-linking antibody in 96 well flat bottom plates. Directly prior transduction 50 µl of the growth medium was removed by pipetting. The LV (40 TU/cell) diluted in 50 µl StemMACS HSC Expansion Medium XF (50 IU/ml IL-4)) was added to the cells. One day post transduction medium was exchanged with 200 µl StemMACS HSC Expansion Medium XF supplemented with 50 IU/ml IL-4, CD40-L with 3.6 µl/ml crosslinking antibody and 5 % AB serum. Transduction efficiency was analyzed 14 days post transduction.

Malignant B cells alone were seeded in a density of 2.66x10^6 cell/ml in 150 µl RPMI (2 mM stable Glutamine, SCF/IL-3/Flt-3). Respective samples were preincubated for 30min with Raltegravir (1µM) and GFP-encoding LV was added (10 TU/cell). The medium of the malignant cells transduced in absence of PBMC was replaced 24 h post transduction with RPMI (20 % FCS, 2 mM stable Glutamine, SCF/IL-3/Flt-3, (Miltenyi Biotec, Bergisch Gladbach, Germany) +/- Raltegravir). 4 days post transduction the cells were stained for viability and surface expression of CD3, CD14, CD16, CD56, CD19, CD20. Transduction efficiency was analyzed by quantification of GFP positive cells. Flow cytometry was performed using the MACSQuant Analyzer 10 (Miltenyi Biotec, Bergisch-Gladbach, Germany) and the data was analyzed with FlowLogic (Inivai Technologies, Mentone Victoria, Australia).

Co-culture transduction with activated T cells

Primary T cells of two healthy donors were isolated one day prior to transduction using the PAN T cell isolation kit (Miltenyi Biotec, Bergisch Gladbach, Germany) and activated by cultivation over night in TexMACS medium supplemented with 12.5ng/ml IL7, 12.5ng/ml IL15 and Transact (Miltenyi Biotec, Bergisch Gladbach, Germany). A mixture of activated T cells of healthy two donors (30 %) and malignant B cells (70 %) were seeded in a density of 2.66x10^6 cell/ml in 150 µl RPMI (2 mM stable Glutamine, SCF/IL-3/Flt-3). Respective samples were preincubated for 30min with Raltegravir (1µM) and GFP-encoding LV displaying anti-CD19 CAR, anti-CD20 CAR, anti-CD318 CAR or no CAR (5 TU/cell) was added. Excess LV was removed by three successive washing steps after 1.5 h at 37°C incubation using RPMI w/o supplements for co-culture transductions. Cells were cultivated in RPMI (20 % FCS, 2 mM stable Glutamine, SCF/IL-3/Flt-3, (Miltenyi Biotec, Bergisch Gladbach, Germany) +/- Raltegravir). 4 days post transduction the cells were stained for viability and surface expression of CD45, CD3, CD14, CD16, CD56, CD19, CD20. Transduction efficiency was analyzed by quantification of GFP positive cells. Flow cytometry was performed using the MACSQuant Analyzer 10 (Miltenyi Biotec, Bergisch-Gladbach, Germany) and the data was analyzed with FlowLogic (Inivai Technologies, Mentone Victoria, Australia).

Production of GALV-RV

GALV pseudotyped RVs were produced by transient transfection of HEK293T cells or HEK-293T cells stably expressing CARs and LNGFR to generate GFP-encoding RV displaying CAR/LNGFR. For this 19.3µg pHIT, 25.7µg pLEGFP-N1 and 9.7µg of pCOLT-GALV. The RV was harvested 48 h post transfection. To remove cellular debris, the supernatant was collected, centrifuged for 10 min at 1000 rpm, followed by filtration through a 0.45 µm filter. To concentrate, the filtered supernatant was centrifuged for 24 h at 4 °C with 5350xg through a 20 % sucrose cushion. The pelleted RV was resuspended in precooled PBS, aliquoted and stored at -80 °C for later use. The transfer plasmids encoding a RV titers were determined by transducing HT1080 cells with serially diluted GFP encoding RV in DMEM (BioWest, Nuaillé, France) without supplements. 72 h post transduction the transduction efficiency was determined by flow cytometry determining the ratio of GFP. The ratio of GFP positive cells, the dilution factor and the volume of retroviral vector particles applied was used to calculate the RV titer (i.e. transducing units per volume (TU/ml)).

Binding Assay with GALV-RV

The protocol described under Material and Methods of the main manuscript was followed. A LV dose of 0.5TU/cell was applied.

In solution digest of lentiviral vector

Purified and concentrated GMP-grade lentiviral vector samples were heated up to 95 °C for 15 min in the presence of 0.05% Rapigest detergent (Waters, Eschborn, Ger). Samples were digested with trypsin overnight at 37 °C and subsequently acidified with 1 µl 10% trifluoracetic acid. C18 Stage tips (Thermo Scientific, Dreieich, Ger) were used to desalt and concentrate peptides. Peptides were eluted with 30 µl 80% acetonitrile and 0.1% formic acid, dried in a speed vac and dissolved in 20 µl 2% acetonitrile and 0.1% formic acid for analysis using mass spectrometry.

Lentiviral vector characterization using LC-MS/MS

LC-MS/MS was carried out using an Ultimate 3000 RSLCnano system coupled to a Thermo Scientific Orbitrap Fusion Lumos mass spectrometer (Thermo Fisher Scientific, Dreieich, Ger). The peptides were desalted in a trapping cartridge (Acclaim PepMap reversed phase C18, 5 µm,100Å, 100 µm x 2 cm length; Thermo Fisher Scientific) and separated on a C18 reversed phase nano-column (2 µm, 100 Å, 75 µm x 15 cm length; Thermo Fisher Scientific) using gradient conditions at a flow rate of 0.3 µl/min (mobile phase A: water with 0.1% formic acid; mobile phase B: acetonitrile with 0.1% formic acid). A step gradient with 2-30% B (5-40 min), 30-55% B (40-45 min) and 55-90% B (45-45.5 min was used with a total LC analysis time of 56 min. Separated and eluted peptides were sprayed at 2.2 kV needle voltage into the mass spectrometer. MS full scans were performed at 350-1500 m/z (mass-to charge-ratio) and a resolution of 120000 in the orbitrap. A top speed method was used with a intensity threshold of 5.0e4 for the peptides selected for MS/MS. The cycle time for each MS/MS was three sec and mass measurement was performed with the resolution of 15,000 in the orbitrap mass analyzer. Peptides were fragmented by collision induced dissociation at collision induced energy of 35% and dynamic exclusion was set to one. Mass spectra processing and database searching was carried out using PEAKS Studio 8.5 (Bioinformatics Solutions Inc., Waterloo, Canada). The precursor mass accuracy was set to 5 ppm and the fragment ion tolerance to 0.05 Da. Carbamidomethylation at cystein, pyro-glu from glutamine, oxidation of methionine, histidine and tryptophan as well as tryptophan oxidation to kynurenin were set as variable posttranslational modifications. For MS/MS peptide sequences up to three missed cleavages per peptide were allowed and the false discovery rate was set to 0.1%. A protein FASTA database including all lentiviral protein sequences of interest was used (Gag, Pol, VSV-G Env, LNGFR, CD19 CAR, CD20 CAR).

Supplemental Figures

Figure S1:

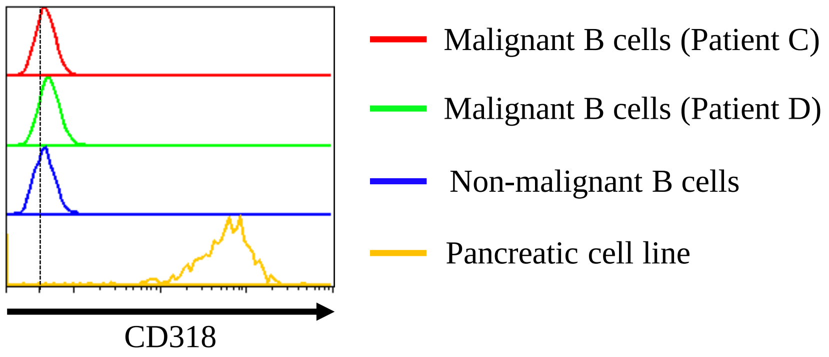

Figure S1. CD318 expression of healthy and malignant B cells.
Absence of expression of the CD318 on primary healthy B cells and primary B-ALL cells was confirmed by staining for CD318 and subsequent flow cytometry analysis. A CD318-expressing pancreatic cell line was used as positive control.

Figure S2:

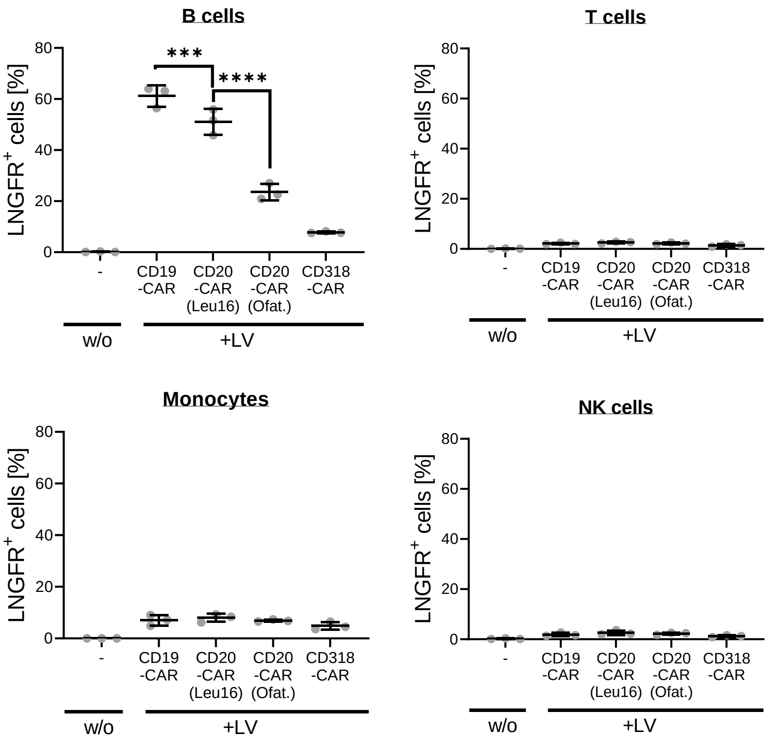

Figure S2. Binding of different CD20-CAR clones to unstimulated PBMC.
Unstimulated PBMC of three healthy donors were left untreated (-) or were incubated with anti-CD19 (clone: FMC63), anti-CD20 (clone:Leu16), anti-CD20 (clone: ofatumumab) or anti-CD318 CAR- and LNGFR-encoding LV (MOI= 10). After binding for 1 h at 4 °C, excess LV was removed by subsequent cell washing and cell-bound LV was detected by staining for LNGFR on the different cellular subsets namely T cells (CD3+, CD56-), Monocytes (CD3-, CD14+) and B cells (CD3-, CD19+). For each donor triplicates were analyzed. The average of triplicates for three donors ± SD is shown. ***P= .0006, ****P< .00001, 2-way ANOVA with multiple comparisons.

Figure S3:

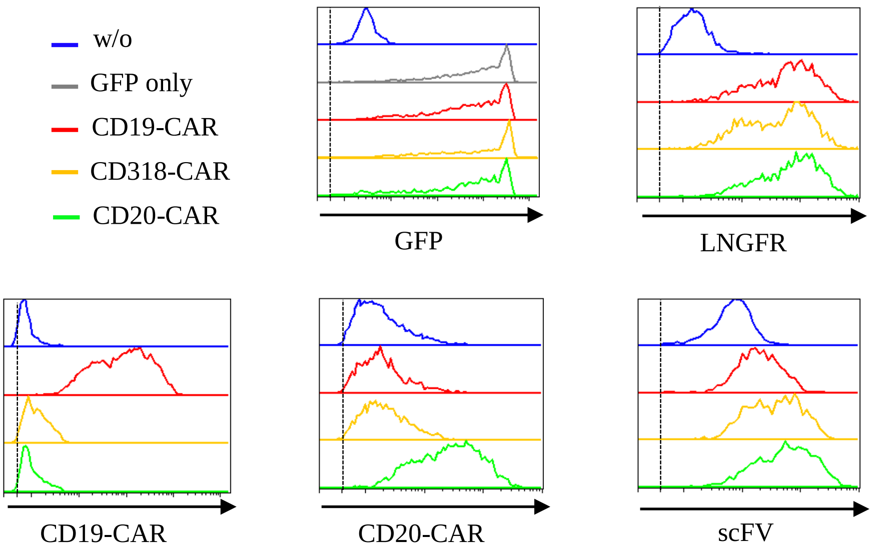

Figure S3. Generation of stably CAR expressing HEK293T cells.
HEK293T cells were genetically engineered by transduction with LVs to stably express an anti-CD19 CAR, anti-CD20 CAR or anti-CD318 CAR and LNGFR. Expression of the transgene was confirmed by staining for LNGFR, anti-CD19 CAR, anti-CD20 CAR or the murine scFV and subsequent flow cytometry analysis. The expression levels of LNGFR of the HEK293T cells expressing either the anti-CD19 CAR, the anti-CD20 CAR or the anti-CD318 CAR was compared to untreated HEK293T cells (w/o). The GFP expression during LV production was analyzed on the day of harvest and compared to HEK293T cells expressing no CAR (GFP only).

Figure S4:

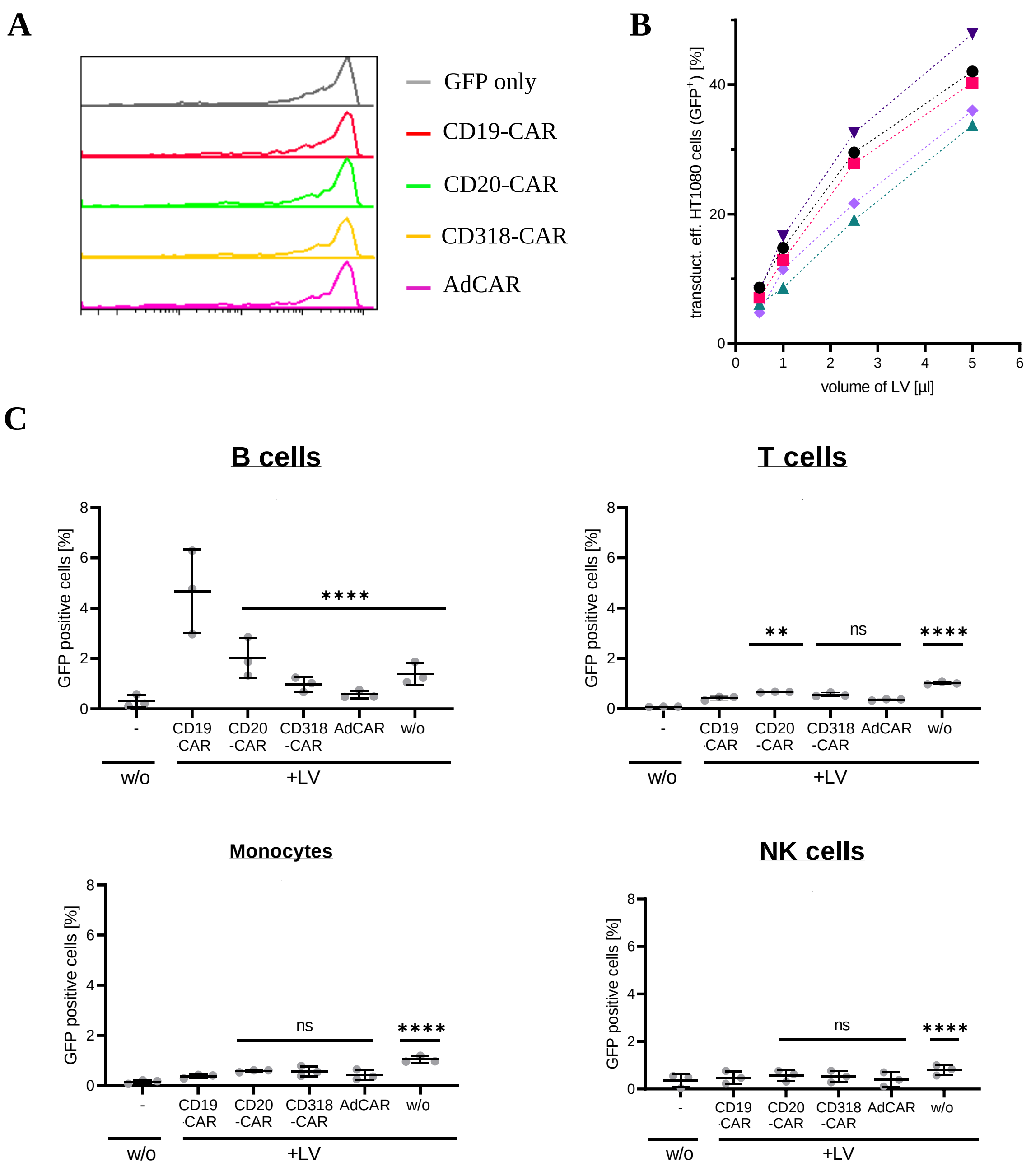

Figure S4. Binding of GALV-RV to unstimulated PBMC.
(**A**) The GFP expression during LV production was analyzed on the day of harvest and compared to HEK293T cells expressing no CAR (GFP only). (**B**) Titration of GALV-RV on HT1080 cells with serially diluted RV. (**C**) Unstimulated PBMC of three donors were left untreated (-) or were incubated with GFP-encoding RVs displaying anti-CD19, anti-CD20 or anti-CD318 CAR or AdCAR or no additional surface protein (w/o) (MOI= 0.5). After binding for 1 h at 4 °C, excess LV was removed by subsequent cell washing and cell-bound RV was detected by staining for LNGFR on the different cellular subsets namely T cells (CD3+, CD56-), Monocytes (CD3-, CD14+) and B cells (CD3-, CD19+). For each donor triplicates were analyzed. The average of triplicates for three donors ± SD is shown. Ns= not significant, ****P< .00001, 2-way ANOVA with multiple comparisons.

Figure S5:

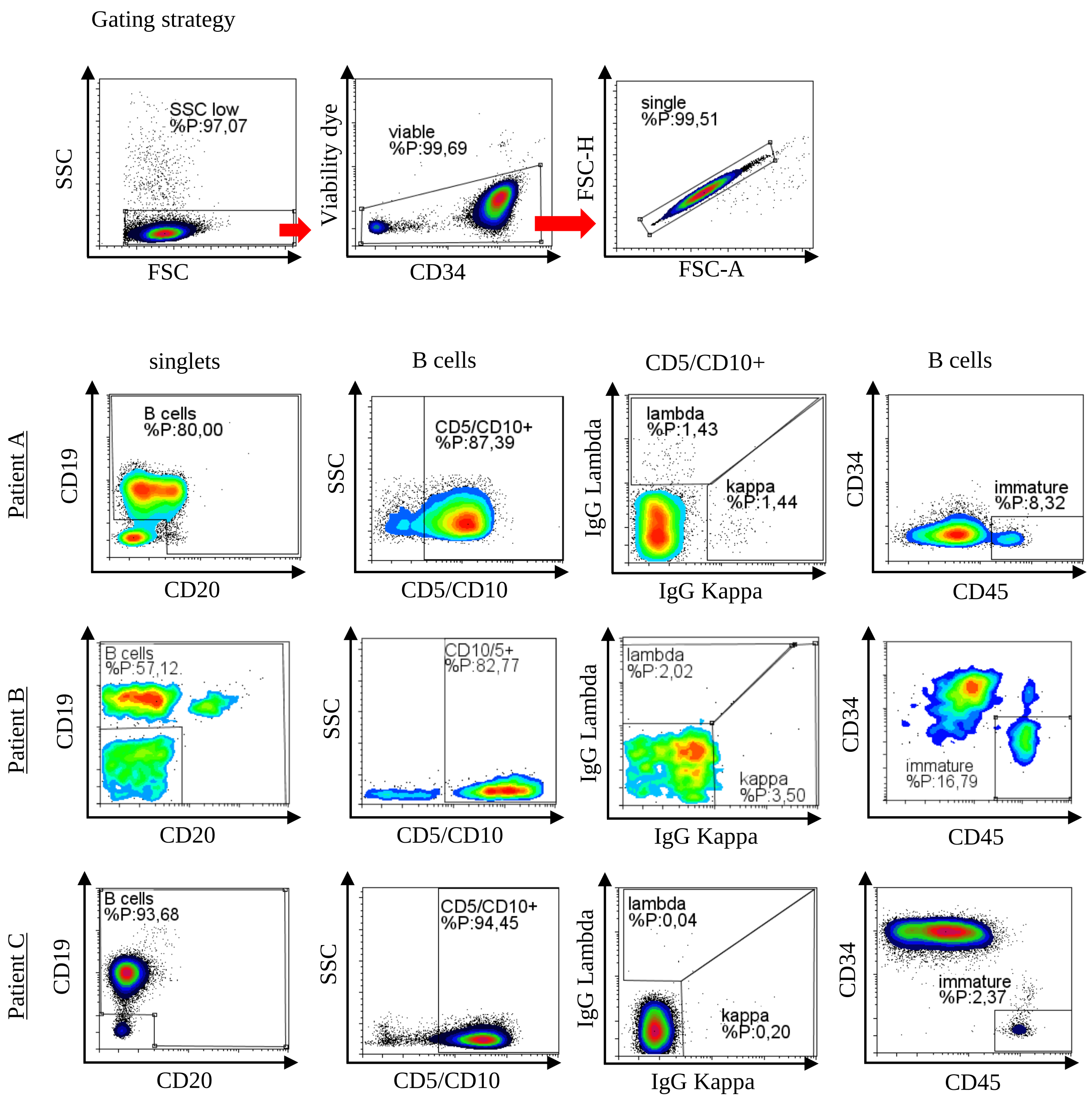

Figure S5. Characterization of primary malignant B cells.
Leukocytes were separated from residual erythrocytes/granulocytes by Ficoll (Pan-Biotech, cat# P04-601000). Obtained cells were stained for CD45, CD19, CD20, CD5, CD10, CD34, IgG Lambda and IgG Kappa and analyzed by flow cytometry. Results for three different B-ALL samples including gating strategy are shown.

Figure S6:

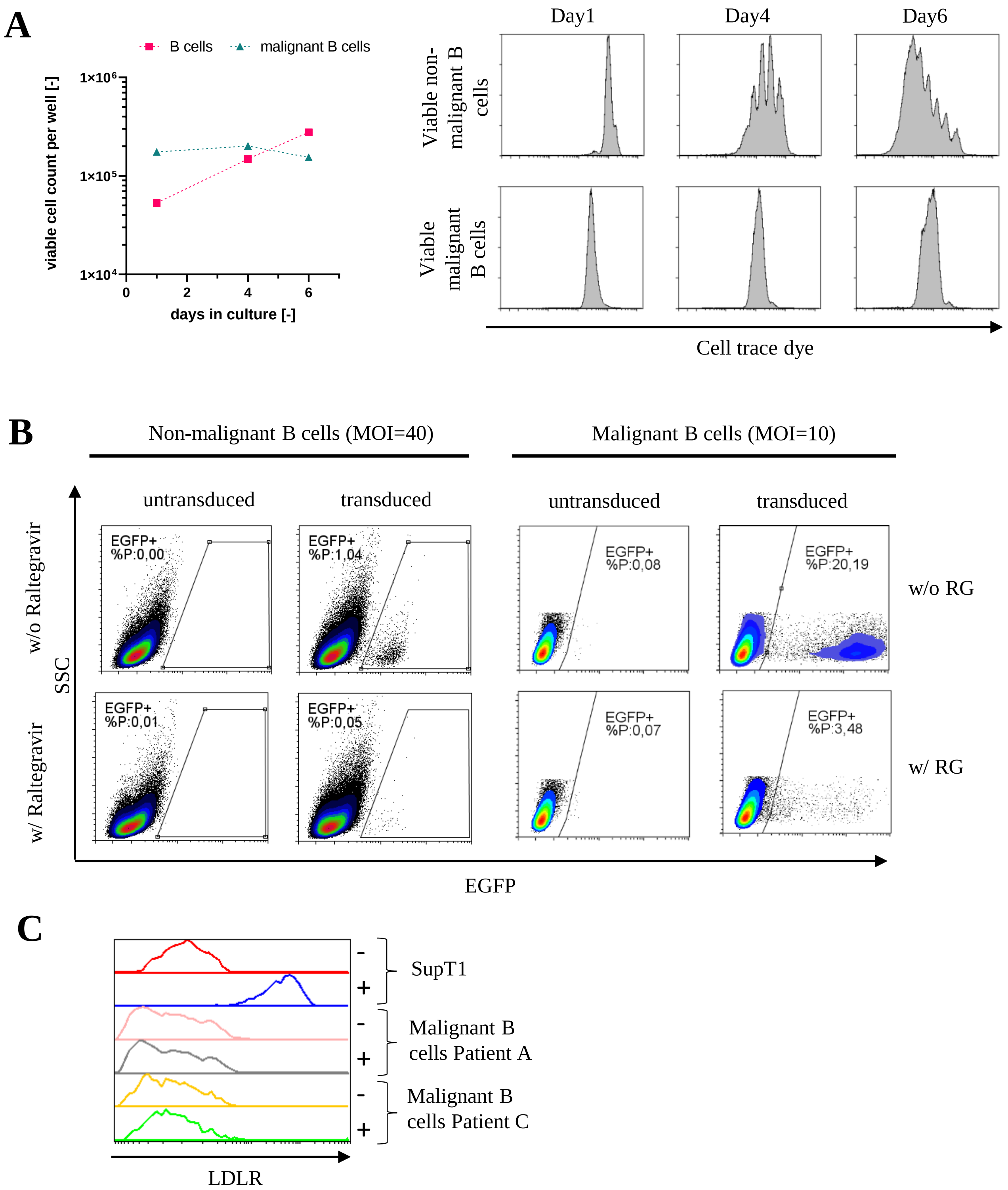

Figure S6. Comparison of transduction of healthy and malignant B cells.

(**A**) Cell proliferation of malignant and non-malignant B cells under chosen cultivation conditions evaluated by counting viable cells using the MACSQuant Analyzer 10 (left) and by following cell division using Violett Cell trace dye (Thermo Fisher Scientific, Waltham, Massachusetts, USA). (**B**) Healthy and malignant B cells were transduced with GFP-encoding LVs in presence (w/ RG) or absence (w/o RG) of the HIV integrase inhibitor Raltegravir. Transduction efficiency was analyzed by quantification of GFP^{+} cells 7 d (healthy B cells) or 5 d (malignant B cells) post transduction. (**C**) LDLR receptor staining on malignant B cells and on SupT1 cells (positive control) (clone: 472413, Thermo Fisher Scientific, Waltham, Massachusetts, USA) . Depicted are the results of staining without first antibody (-) as negative control and with both antibodies (+).

Figure S7:

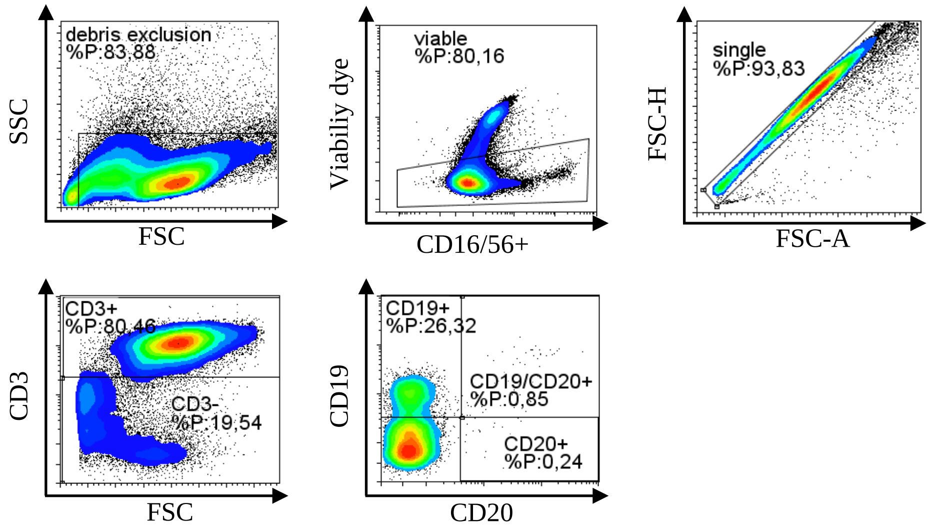

Figure S7. Gating strategy for analysis of transduction of primary malignant B cells.
The gating strategy for flow cytometry analysis of transduced malignant B cells in co-culture with healthy PBMC is shown. In a first step, cell debris was excluded. Next, viable and single cells were identified, and separated in CD3+ and CD3- cells. CD3- cells were separated in CD19+ and CD20+ populations. Transduction efficiency was analyzed on CD19+/CD20- cells.

Figure S8:

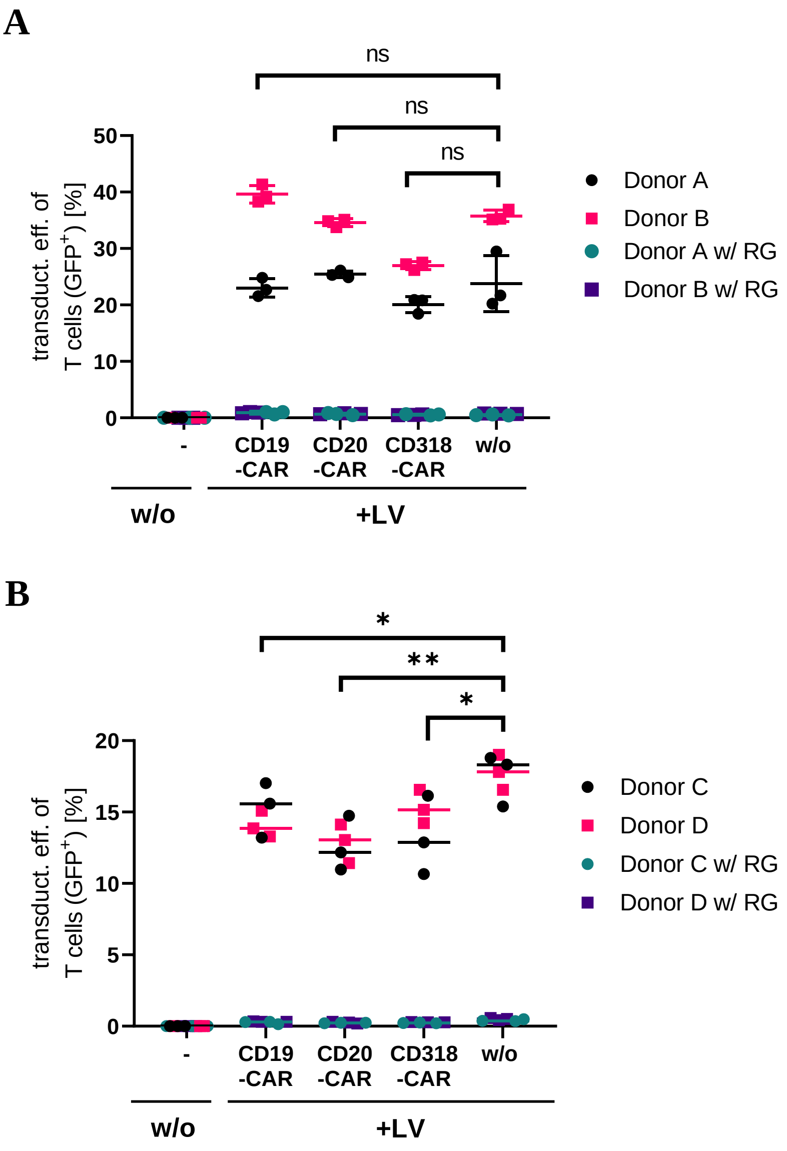

Figure S8. Transduction of activated T cells with GFP-encoding LVs displaying different CARs of different specificities and LNGFR.

Pan T cells were isolated from PBMC of two healthy donors using negative magnetic selection (Miltenyi Biotec, cat# 130-096-535). T cells were activated with Transact® (Miltenyi Biotec, cat# 130-111-160) overnight, respective samples were treated with the integrase inhibitor Raltegravir (RG) and transduced with GFP-encoding LVs displaying either the anti-CD19-, anti-CD20-, anti-CD318- or no CAR (w/o) in triplicates. Transduction efficiency was analyzed 4 d post transduction by quantification of the GFP positive cells by flow cytometry. (**A**) Analysis of the LV batch used for the experiments of Figure 2 and Figure 4. ns, not significant (P= .999 [CD19-CAR/w/o], P= .999 [CD20-CAR/w/o], P= .999 [CD318-CAR/w/o]). (**B**) Analysis of the LV batch used for the experiments of Figure 3 and Figure 5. *P (P= .0476 [CD19-CAR/w/o], P= .0285 [CD318-CAR/w/o]), **P .0057 . Ordinary one-way ANOVA with multiple comparisons.

Figure S9:

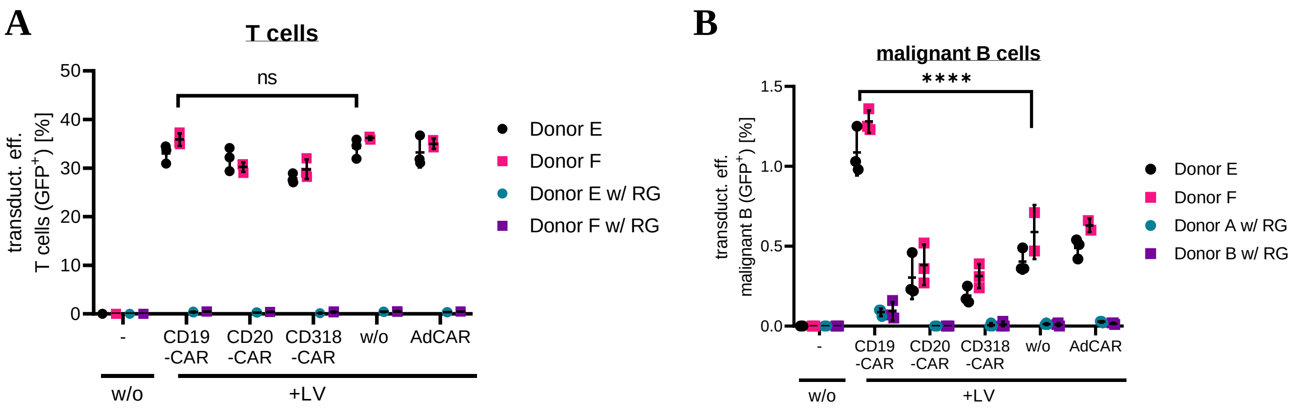

Figure S9. Transduction of malignant B cells in presence of activated T cells.
70% malignant B-precursor cells of one B-ALL patient were mixed to 30% activated T cells of two healthy donors. GFP-encoding LVs displaying different CAR proteins and LNGFR were added for 90 min at 37 °C (MOI 5). Unbound LV was removed by four successive washing steps and the transduction efficiency was analyzed 4 days post transduction by flow cytometry. Triplicates from one experiments ± SD are shown. ****P< .0001, ns= not significant P=.9809, ordinary two-way ANOVA.

Figure S10:

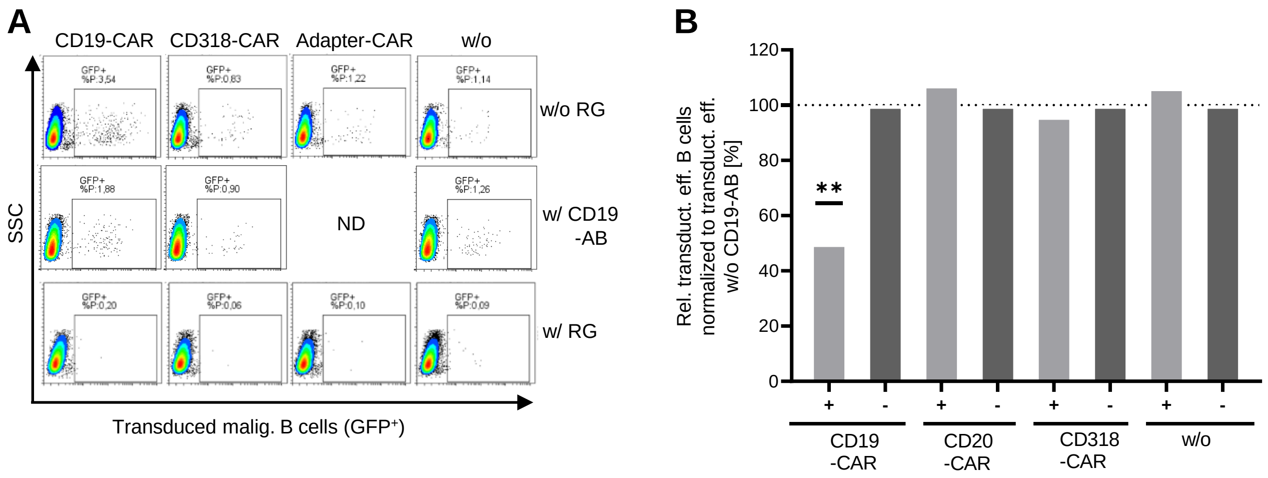

Figure S10. Transduction of malignant B cells with GFP-encoding LVs displaying CARs of different specificities and LNGFR.

30% malignant B-precursor cells of one B-ALL patient were mixed to 70% PBMC of a healthy donor. LV (MOI 40) and the LV-inhibitor Raltegravir were added to respective samples. Additional samples were pretreated with CD19-blocking antibody (1µg/ml) for 30min at 4°C (w/ CD19-AB). GFP-encoding LVs displaying different CAR proteins and LNGFR were added for 90 min at 37 °C. Unbound LV was removed by three successive washing steps and the transduction efficiency was analyzed 4 days post transduction by flow cytometry. (**A**) Transduction of malignant B cells co-cultured with PBMC is shown (representative data of triplicates of one donor). Respective samples were treated with Raltegravir (w/ RG) or left untreated (w/o RG). (**B**) Transduction of B cells in presence or absence of CD19-AB normalized to transduction in absence of antibody. **P= .0024, student t test. ND, not determined

Supplemental Tables

Table S1. Coverage of viral protein sequences by LC-MS/MS in purified GMP-grade LV preparations.

Viral/CAR protein		Protein length (amino acids)	CD19-CAR LV			
			**Lot 1**	**Lot 2**	**Lot 3**	**Lot 4**
**Gag**	MA (matrix protein)	130	76%	70%	67%	76%
	CA (capsid protein 24)	230	79%	76%	46%	88%
	p2	15	ns	ns	ns	ns
	p6	51	100%	100%	100%	100%
**Pol**	PR (Peptidase A2)	69	69%	69%	69%	69%
	RNase H	123	51%	41%	ns	60%
	RT (reverse transcriptase)	190	54%	46%	36%	55%
	IN (integrase)	150	22%	30%	20%	27%
**VSV-G Env**		511	52%	53%	39%	61%
**CD19-scFV**	CD19 light chain	107	22%	29%	22%	31%
	CD19 heavy chain	131	ns	ns	ns	21%
**CAR**	CD8 transmembrane domain	72	14%	14%	14%	14%
	41BB costim	42	ns	ns	ns	14%
	CD3 zeta domain	112	89%	88%	88%	91%

Gag: group-specific antigen, Pol: polymerase, Env: envelope, CAR: chimeric antigen receptor, LV: lentiviral vector
