# Supplementary material for: Anti-CD19 CARs displayed at the surface of lentiviral vector particles promote transduction of target-expressing cells
Source: Mol Ther Methods Clin Dev. 2021 Feb 24;21:42–53. doi: 10.1016/j.omtm.2021.02.013 (PMC7966970; doi:10.1016/j.omtm.2021.02.013)
Supplement: Document S2. Article plus supplemental information [file mmc2.pdf]

Molecular Therapy
Methods & Clinical Development
Original Article

Anti-CD19 CARs displayed at the surface of lentiviral vector particles promote transduction of target-expressing cells

Nicole Cordes,[1,2] Carolin Kolbe,[1] Dominik Lock,[1] Tatjana Holzer,[1] Deborah Althoff,[1] Daniel Schäfer,[1] Franziska Blaeschke,[3] Bettina Kotter,[1] Sandra Karitzky,[1] Claudia Rossig,[4] Toni Cathomen,[5,6] Tobias Feuchtinger,[3] Iris Bürger,[1] Mario Assenmacher,[1] Thomas Schaser,[1,7] and Andrew D. Kaiser[1,7]

[1]Miltenyi Biotec B.V. & Co. KG, 51429 Bergisch Gladbach, Germany; [2]Faculty of Biology, University of Freiburg, 79104 Freiburg, Germany; [3]Department of Pediatric Hematology, Oncology, Hemostaseology, and Stem Cell Transplantation, Dr. von Hauner Children's Hospital, University Hospital, LMU Munich, 80337 Munich, Germany; [4]Department of Pediatric Hematology and Oncology, University Children's Hospital Muenster, 48149 Muenster, Germany; [5]Institute for Transfusion Medicine and Gene Therapy, Medical Center - University of Freiburg, 79106 Freiburg, Germany; [6]Faculty of Medicine, University of Freiburg, 79110 Freiburg, Germany

Recently, a rare type of relapse was reported upon treating a B cell acute lymphoblastic leukemia (B-ALL) patient with anti-CD19 chimeric antigen receptor (CAR)-T cells caused by unintentional transduction of residual malignant B cells (CAR-B cells). We show that anti-CD19 and anti-CD20 CARs are presented on the surface of lentiviral vectors (LVs), inducing specific binding to the respective antigen. Binding of anti-CD19 CAR-encoding LVs containing supernatant was reduced by CD19-specific blocking antibodies in a dose-dependent manner, and binding was absent for unspecific LV containing supernatant. This suggests that LVs bind via displayed CAR molecules to CAR antigen-expressing cells. The relevance for CAR-T cell manufacturing was evaluated when PBMCs and B-ALL malignant B cells were mixed and transduced with anti-CD19 or anti-CD20 CAR-displaying LVs in clinically relevant doses to mimic transduction conditions of unpurified patient leukapheresis samples. Malignant B cells were transduced at higher levels with LVs displaying anti-CD19 CARs compared to LVs displaying non-binding control constructs. Stability of gene transfer was confirmed by applying a potent LV inhibitor and long-term cultures for 10 days. Our findings provide a potential explanation for the emergence of CAR-B cells pointing to safer manufacturing procedures with reduced risk of this rare type of relapse in the future.

INTRODUCTION

The success of adoptive immunotherapy using anti-CD19 chimeric antigen receptor (CAR)-expressing T cells to treat B cell acute lymphoblastic leukemia (B-ALL), though remarkable, is limited by a considerable number of relapses attributed to antigen loss.[1] Recently, an additional type of CAR-T cell resistance has been reported, caused by the unintended transduction of residual malignant B cells with anti-CD19 CAR-transferring lentiviral vectors (LVs) causing antigen masking and relapse.[2] Thus, residual malignant B cells present during the transduction represent a risk for CAR-T cell manufacturing. To provide solutions that improve the safety of CAR-T cell manufacturing, it is crucial to shed light into the mechanisms involved in the unintentional transduction of malignant B cells.

Antigen escape caused by the unintentional transduction of malignant B cells was discovered upon treating a patient with tisagenlecleucel, a drug product based on the transduction of activated peripheral blood mononuclear cells (PBMCs) with VSV-G pseudotyped LVs.[3,4] LVs are typically pseudotyped with VSV-G to induce viral entry into a broad range of cells expressing the low-density lipoprotein receptor (LDLR) and related family members. While investigating the transduction of PBMCs with CAR-encoding VSV-G LVs, we detected CAR protein on transduced cells directly after LV addition. Since this observation did not match the known kinetics of transgene expression, the CAR protein must have been transferred via the LV-containing supernatant. Associated impurities, VSV-G vesicles, or lentiviral particles represent potential sources of this protein transfer.[5] The LV membrane is derived from the packaging cells; hence, it may also contain host cell proteins.[6,7] Incorporation of host cell proteins into human immunodeficiency virus (HIV) particles and lentiviral vectors has been described before. Their level of incorporation is highly variable and dependent on the specific protein: some proteins are enriched (e.g., ICAM-1), while others are excluded (e.g., CD4, CD27).[5,7,8] Expression of heterologous proteins was also observed to result in efficient protein incorporation into the LV membrane (e.g., CD20 or low-affinity nerve growth factor receptor (LNGFR)). Expression of such heterologous proteins can be used as markers to detect cell-bound LVs.[9–11]

Received 20 August 2020; accepted 19 February 2021;
https://doi.org/10.1016/j.omtm.2021.02.013.

[7]Senior author

Correspondence: Thomas Schaser, Miltenyi Biotec B.V. & Co. KG, Friedrich-Ebert-Straße 68, 51429 Bergisch Gladbach, Germany.
E-mail: thomasscha@miltenyi.com

 © 2021 The Author(s).
This is an open access article under the CC BY license (http://creativecommons.org/licenses/by/4.0/).

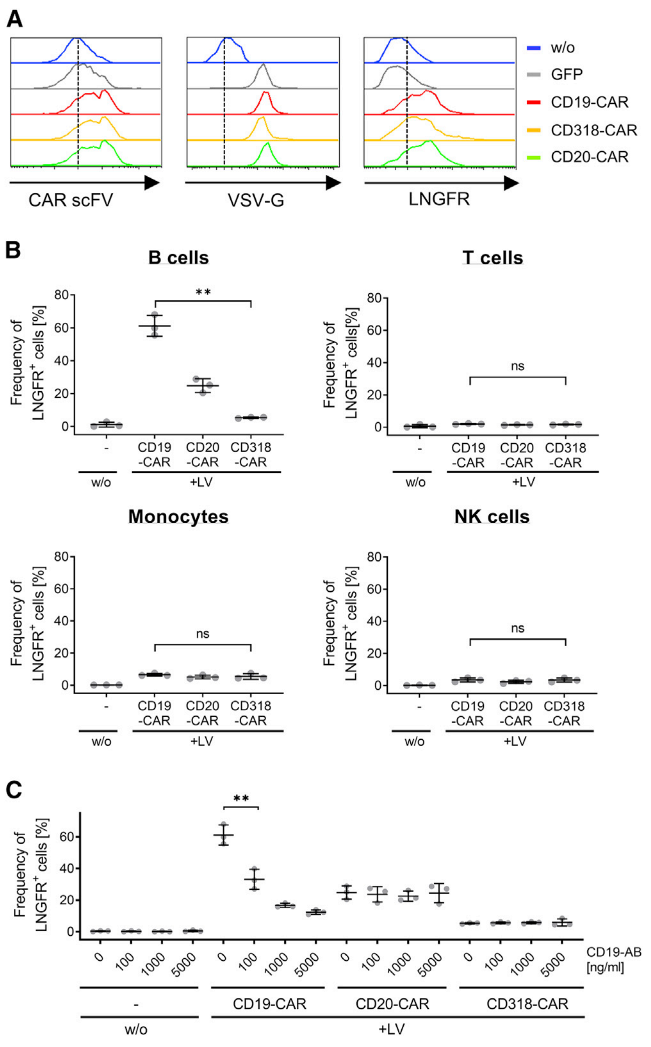

Figure 1. CARs are present within LV preparations and mediate binding to CAR antigen-expressing cells

(A) SupT1 cells were left untreated (w/o) or were incubated with supernatant containing anti-CD19, anti-CD20, anti-CD318 CAR, or GFP-encoding LVs. Cell-bound particles were detected by staining for LNGFR, VSV-G, or the scFV. (B) Unstimulated PBMCs of three healthy donors were left untreated (–) or were incubated with supernatant containing anti-CD19, anti-CD20, or anti-CD318 CAR-encoding LVs. Cell-bound particles were detected by staining for LNGFR on the different cellular subsets, namely T cells (CD3+, CD56–), Monocytes (CD3–, CD14+), and B cells (CD3–, CD19+). For each donor, triplicates were analyzed. The average of triplicates for three donors ± SD is shown. **p = 0.0042, unpaired two-tailed t test with Welch correction. ns, not significant (p = 0.2338 [T cells], p = 0.378 [monocytes], p = 0.96 [NK cells]); unpaired two-tailed t test of mean value of three donors. (C) Unstimulated PBMCs of three donors were preincubated with a CD19-specific antibody ranging from 0–5,000 ng/mL followed by incubation with supernatant containing LVs encoding anti-CD19, anti-CD20, or anti-CD318 CAR. Cell-bound particles were detected on B cells (CD3–, CD19+, CD20+) by staining for LNGFR. The average of triplicates for three donors ± SD are shown. **p = 0.0057, unpaired two-tailed t test of mean value of three donors.

To evaluate the source of protein transfer, we performed binding studies with LVs containing supernatant, which showed that anti-CD19 (FMC63-derived) and anti-CD20 CAR-encoding LVs efficiently bind to B cells. This finding led us to hypothesize that CARs are displayed on the LV surface and that the antigen specificity of the CAR contributes to the specificity of LV binding. Finally, to determine the clinical relevance in the context of B-ALL, we evaluated whether CAR display alters the tropism of VSV-G pseudotyped LVs toward increased transduction efficiencies on malignant B cells.

RESULTS

CARs are displayed at the surface of LVs and mediate binding to the respective antigen

Detection of CAR protein directly after adding LV prompted us to investigate the source of the protein transfer. Since LV supernatant cannot be directly analyzed by flow cytometry, we evaluated the presence of CAR protein by staining particles bound to SupT1 cells expressing the VSV-G receptor LDLR. Binding was performed at 4°C to prevent membrane fusion activity of VSV-G, LV entry, and loss of detectable LV.[12] CAR protein in viral supernatant was found at low but detectable levels (Figure 1A). This finding was surprising, as published studies could only find low levels of CAR protein by western blot but fail to detect CAR protein in their LV preparations by flow cytometry.[9] At this point, the source of CAR protein transfer cannot be identified, as impurities such as free protein or extracellular vesicles and lentiviral particles may bind to SupT1 cells.

Thus, to evaluate if CAR protein can also be found in purified LV preparations of higher quality, GMP-grade LVs encoding a clinically relevant anti-CD19 CAR were analyzed by liquid chromatography-tandem mass spectrometry (LC-MS/MS) (Table S1). All relevant viral proteins, but more importantly the different domains of anti-CD19 CAR, were detected in four different LV batches. This confirmed the presence of CAR protein in clinical-grade LV preparations.

To investigate whether the detected CAR protein is also displayed at the particle surface and retained target binding function, we performed binding studies at 4°C on unstimulated PBMCs using preparations containing anti-CD19, anti-CD20, or anti-CD318 CAR-encoding LVs (Figure 1B). While CD19 and CD20 are expressed on B cells, CD318 is not described to be expressed on PBMCs. Absence of CD318 on both non-malignant and malignant B cells was confirmed by staining with CD318-specific antibodies and subsequent flow cytometry analysis (Figure S1).

Unstimulated PBMCs were used to reduce particle binding via VSV-G to its receptor LDLR.[13] Here, LNGFR, which was co-expressed with the CAR, was used as detection marker for viral particles (Figures 1B and 1C). After removing excess particles by washing, cell-bound particles were measured by LNGFR staining among T cells (CD3+, CD56–), monocytes (CD3–, CD14+), natural killer (NK) cells (CD3–, CD56+), and B cells (CD3–, CD19+/CD20+). LNGFR-positive B cells were detectable at more than 10-fold higher levels for

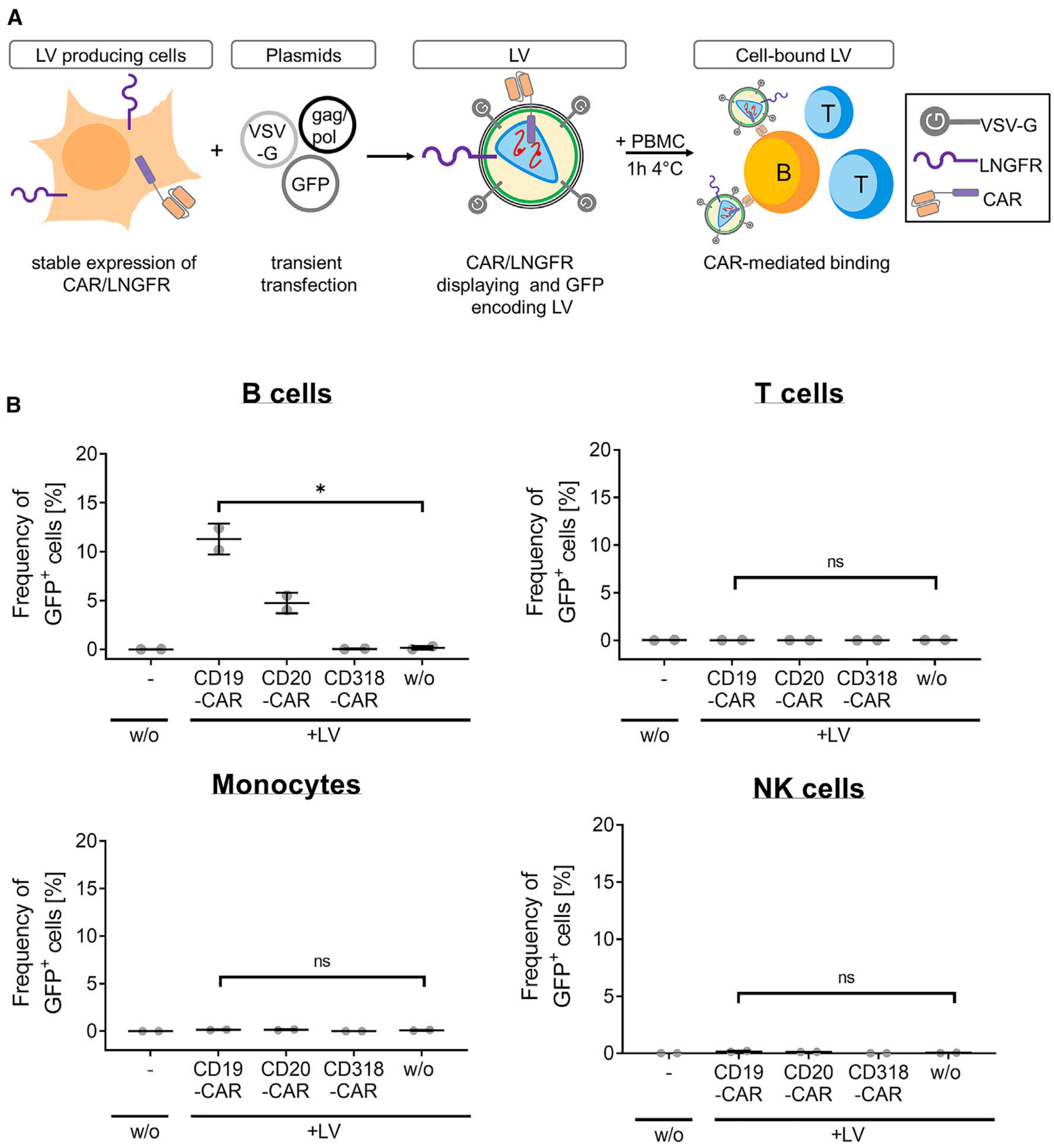

Figure 2. GFP-encoding and CAR-displaying particles within LV preparations are binding to CAR antigen-expressing cells

(A) A schematic representation of the experimental setup is shown. HEK293T cells were genetically engineered by transduction with LVs to stably express an anti-CD19 CAR, anti-CD20 CAR, or anti-CD318 CAR and LNGFR. GFP-encoding LVs were generated from CAR/LNGFR-expressing HEK293T cells. GFP was used as readout parameter to determine the frequency of cells with bound LV. (B) Unstimulated PBMCs of two donors were left untreated (–) or were incubated with supernatant containing GFP-encoding LVs displaying anti-CD19, anti-CD20, or anti-CD318 CAR and LNGFR or no additional surface protein (w/o). Cell-bound particles were detected by quantification of GFP^+ cells on the different cellular subsets, namely T cells (CD3+, CD56–), monocytes (CD3–, CD14+), and B cells (CD3–, CD19+). The mean of triplicates for two donors ± SD are shown. *p = 0.0102; ns, not significant (p = 0.7885 [T cells], p = 0.3451 [monocytes], p = 0.0914 [NK cells]); unpaired two-tailed t test.

supernatant containing anti-CD19 CAR-encoding LV as compared to anti-CD318 CAR-encoding LV supernatant. For anti-CD20 CAR LV-containing supernatant, a 4.5-fold higher level of particle-bound B cells was observed as compared to anti-CD318 CAR-encoding LV-containing supernatant.

Antigen-blocking experiments with antibodies were performed to exclude unspecific binding. Notably, CD19-specific antibodies induced a concentration-dependent inhibition of particle binding for anti-CD19 CAR-encoding LV supernatant only (Figure 1C). In order to test whether this phenomenon is also epitope dependent,

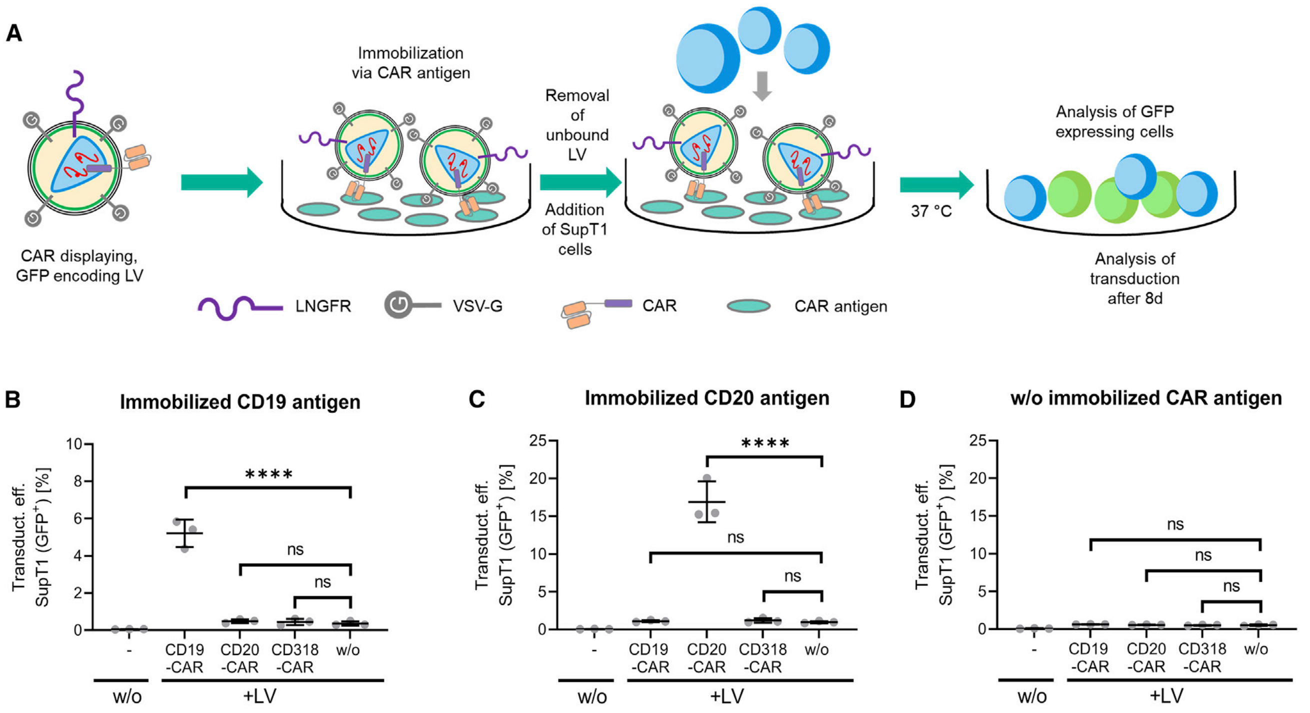

Figure 3. CAR display mediated binding of LV to the respective CAR antigen

(A) A schematic representation of the experimental setup is shown. GFP-encoding LVs displaying anti-CD19, anti-CD20, or anti-CD318 CAR and LNGFR or no additional surface protein (w/o) were added to the wells of an ordinary ELISA plate that contained immobilized anti-CD19 CAR-detection reagent, CD20 peptide or no antigen, respectively. LV was added for 1 h to enable binding. Unbound LV was removed by multiple washing and SupT1 cells were seeded onto the immobilized particles. Functional LV particles were detected by analysing the transduction efficiency of SupT1 cells expressing GFP 8 days post transduction. (B) Results of transduction upon CD19-specific immobilization of the LV. ****$p < 0.0001$, ordinary two-way ANOVA with multiple comparisons. Data are represented as mean ± SD. (C) Results of transduction upon CD20-specific immobilization of the LV. ****$p < 0.0001$, ordinary two-way ANOVA with multiple comparisons. ns, not significant. Data are represented as mean ± SD. (D) Result of transduction of SupT1 cells when no antigen was immobilized. ns, not significant; ordinary two-way ANOVA with multiple comparisons. Data are represented as mean ± SD.

we evaluated our finding by testing an additional anti-CD20 CAR derived from the well-characterized antibody, ofatumumab, that targets an alternative epitope. For this, the binding experiment was repeated as described before with supernatant containing LV coding for two different anti-CD20 CAR constructs. Both CAR constructs contained an identical extracellular spacer, transmembrane, and cytoplasmic domain but were derived from two different antibody clones, Leu16 and ofatumumab, respectively. As expected, binding with supernatant containing LV encoding ofatumumab-derived CAR resulted in 3-fold higher levels of LNGFR-positive cells compared to anti-CD318 CAR-encoding LV supernatant (Figure S2). Thus, a similar binding pattern was seen when CARs were applied that bind alternative epitopes. Interestingly, differences in frequency of particle-bound B cells were detectable with 2.2-fold lower binding for CARs containing scFVs derived from ofatumumab compared to LVs encoding Leu16-derived CARs. When all B cell-specific CARs are compared, the frequency of particle-bound B cells was highest for CD19 (FMC63)-derived CARs, followed by Leu16-derived CARs and ofatumumab CARs. In summary, although particle binding was observed for all B cell-specific CARs, the frequency of particle binding was influenced by the targeted antigen and the epitope.

These results led us to hypothesize that binding-competent CAR proteins may be incorporated into LV membranes and mediate binding to CAR target antigen-expressing cells. To further evaluate this hypothesis, we aimed to generate LVs that are detectable by GFP upon transduction to simplify a reliable comparison between LVs displaying different CAR constructs. Thus, GFP-encoding LVs were generated that display LNGFR and either anti-CD19, anti-CD20, or anti-CD318-specific CAR. For this, not wild-type HEK293T cells but engineered packaging cells stably expressing the respective CAR construct along with LNGFR were transiently transfected with all the plasmids required for the generation of GFP-encoding LVs. LVs packaged from those cell lines may display CARs on the LV surface but induce GFP expression upon transduction (Figure 2A). Comparable transgene expression levels of the generated cell lines were shown by staining with anti-CD19 CAR and anti-CD20 CAR detection reagents or by staining for LNGFR and murine scFV by staining with an anti-mouse immunoglobulin G (IgG)-specific antibody (Figure S3). Moreover, comparable GFP expression levels during LV production were confirmed. Like LNGFR, GFP protein from the producer cell line is also packaged and was therefore used for detecting cell-bound particles.[14] Since GFP expression can be readily detected without the need of additional staining, potential differences in the

particle-binding capacity caused by staining artifacts were excluded. Similarly, the proportion of B cells with cell-bound particles as measured by GFP was increased 65-fold when treated with anti-CD19 CAR-displaying particles and 28-fold for anti-CD20 CAR-displaying particles compared to particles displaying anti-CD318 CAR or no CAR (Figure 2B). Interestingly, the amount of transferred protein was decreased with GFP-encoding particles as compared to LNGFR-transferring CAR-encoding particles. This was most likely caused by lower CAR/LNGFR expression of the stable producer cells resulting in lower levels of CAR display on the surface of LVs (Figure 1B versus Figure 2B). In contrast, CAR-encoding LVs were generated by transient transfection with plasmids providing multiple copies of CAR cDNA inducing high CAR expression levels. Together, these data suggest that LV preparations produced from cell lines stably expressing CAR also contain CAR protein and mediate binding to CAR target antigen-expressing cells. Importantly, a similar binding pattern for GFP-encoding//CAR-displaying LVs was obtained compared to CAR-encoding/CAR-displaying LVs. Thus, all following experiments were performed using this type of LV.

Neither the binding experiments nor LC-MS/MS analysis allow discrimination between functional and non-functional LVs or extracellular vesicles, which theoretically display CAR molecules on their surface. In addition, binding of LVs was analyzed on PBMC—a mixture of multiple cell types expressing a complex repertoire of surface expression markers. Hence, an assay has been developed that investigates both aspects in combination: the physical interaction between CAR antigen and CAR displayed on LVs and the functionality of bound particles. CAR antigen was immobilized in ELISA plates, and then CAR-displaying, GFP-encoding LV preparations were added. The function of the immobilized particles was evaluated after multiple washing in a subsequent transduction assay by adding SupT1 cells (Figure 3A). 8 days post seeding, functional LVs that specifically bound to the CAR antigen were detected by quantifying transduced SupT1 cells expressing GFP. Immobilization of anti-CD19 CAR and anti-CD20 CAR-displaying LVs via the CAR antigen resulted in a 12-fold and 15-fold increased transduction efficiency on SupT1 cells compared to LVs displaying anti-CD318 or no CAR (Figures 3B and 3C). In contrast, only low transduction efficiencies were observed in the absence of CAR antigen. This confirms that CARs are displayed on functional LVs and that displayed CARs and CAR antigen directly interact (Figure 3D).

Besides lentiviruses, gamma-retroviral vectors (RVs) are also used to genetically engineer T cells for immunotherapy applications. Therefore, the transferability of the mechanism to gibbon ape leukemia virus (GALV) RVs was evaluated briefly. As for lentiviral vectors, GALV-RVs were produced by transient transfection of HEK293T cells that were stably expressing the respective CAR construct. This way, RVs were encoding GFP and displaying the CARs on their surface so that similar observations could be made as with the lentiviral vectors. Subsequently, the binding experiment was repeated according to the protocol applied for Figure 2 by incubating PBMCs from healthy donors with RV-containing supernatant. The data revealed that treatment with GALV-RV supernatant produced from HEK293T cells stably expressing anti-CD19 CAR resulted in 3.5-fold higher frequency of particle-bound B cells compared to supernatant obtained from HEK293T without CAR display (Figure S4). In general, the frequency of particle-bound B cells was lower for all GALV-RV constructs than for the VSV-G-LV counterpart. This was most likely due to the lower maximum dose of GALV-RV we could apply in our experiments. Nevertheless, the observed binding pattern to B cells suggests that the mechanism we describe for VSV-G LV in principle also applies for GALV-RV. However, it is conceivable that the proliferation status may influence the transduction efficiency of RVs.

CAR-mediated binding of LVs promotes transduction of malignant cells

The relevance of CAR display and binding to CAR antigen-expressing cells was evaluated in more detail by transducing a sample containing malignant B cells from three B-ALL patients. The cellular composition was determined by staining for surface expression of CD45, CD19, CD20, IgG kappa, IgG lambda, CD34, CD5, and CD10 (Figure S5).

Although B-ALL cells readily proliferate *in vivo*, *ex vivo* cultivation of B-ALL cells is difficult and mainly supporting cell survival with minimal or even lack of proliferation.[15,16] The cultivation conditions for malignant cells were chosen based on established protocols and were confirmed to enable survival of the cells for the duration of the experiment (Figure S6A).[17] In line with published studies, malignant B cells were transduced at higher levels than B cells of healthy donors—even when a 4-fold lower LV dose was applied (Figure S6B).[17–20] Transduction levels are similar to results published by Biagi and colleagues,[17] who reported a transduction level of 4.4%–21% using a comparable LV dose. In general, transduction efficiency is dependent on multiple factors (e.g., expression of respective surface receptors, cell cycle, and proliferation, as well as cellular restriction factors).[21] To identify the cause of enhanced transduction efficiency of the malignant cells compared to the healthy B cells, we analyzed differences in proliferation capacity by using cell trace dye (Figure S6A). As expected, a lack of proliferation of the B-ALL cells was observed. In contrast, activated healthy B cells readily proliferate under the chosen cultivation conditions, excluding different proliferative capacity as potential cause of increased transduction efficiency. In 2014, LDLR was identified as the main receptor of VSV-G, although related family members of LDLR may be used as well.[12,13] Stimulation via the B cell receptor (BCR) on B cells was shown to only marginally enhance expression of LDLR, which is therefore suspected to cause low transduction levels with VSV-G LV.[13,22] For this reason, we hypothesized that higher expression levels of LDLR on malignant B cells were causing the enhanced transduction efficiency. However, LDLR was not expressed by the malignant cells, as measured by flow cytometry (Figure S6C). The presence of functional LV as measured by transducing activity was confirmed by analyzing control transductions containing raltegravir, an HIV integrase inhibitor, blocking transgene

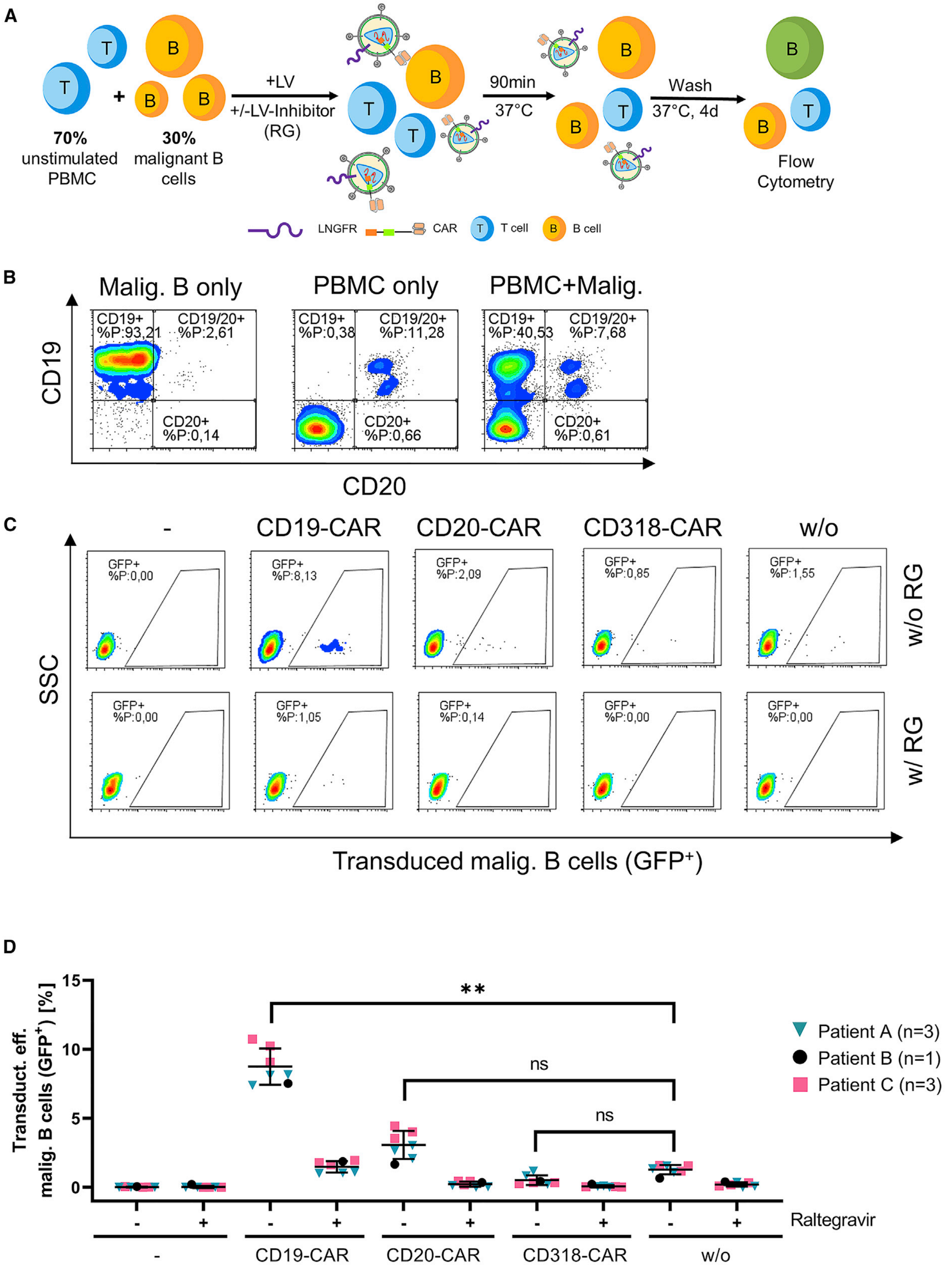

(legend on next page)

integration into the host cell genome and LV-mediated gene expression only.[23] Functional LVs promote raltegravir-dependent, stable expression at high levels. In contrast, exosomes cause transient, highly variable protein transfer (i.e., pseudotransduction) in a raltegravir-independent manner.

Next, we aimed to resemble the cellular composition of a directly transduced leukapheresis sample of a B-ALL patient by transducing a mixture containing 30% malignant B cells and 70% unstimulated PBMCs of a healthy donor (Figure 4A). Malignant B cells (CD19+/CD20−) could be distinguished from residual non-malignant B cells (CD19+/CD20+) solely by analyzing CD19 and CD20 surface expression (Figure 4B; Figure S7). The use of GFP-encoding LVs displaying CARs had multiple advantages: (1) Since GFP is expressed instead of CAR, CD19 antigen is not masked by the CAR, which still allows identification of the cocultured malignant cells by flow cytometry. (2) Applying GFP-encoding LVs further avoids generation of CAR-expressing T cells, which would induce lysis of the malignant B cells. (3) Potential differences in transduction efficiency levels caused by different transfer vector constructs are excluded. LV stocks were titrated by transduction of SupT1 cells with serially diluted LVs and afterwards tested on activated T cells in advance to confirm that the identical LV dose was applied (Figure S8A). Unbound LVs were removed by three successive washing steps 1.5 h post transduction. The transduction efficiency was assessed 4 days later by flow cytometry. Malignant B cells were transduced at 8-fold higher levels when LVs displaying anti-CD19 CARs were applied as compared to LVs displaying anti-CD318 CAR or no CAR. Also, for anti-CD20 CARs displaying LVs, a tendency of increased transduction efficiency was detectable, pointing to malignant cells potentially expressing CD20 at low levels (Figures 4C and 4D). Alternatively, residual LVs still being present after washing may have caused the increased transduction efficiency levels. Importantly, the results were again raltegravir dependent, confirming lentiviral gene transfer and excluding pseudotransduction. In contrast to clinical manufacturing, the culture conditions and the use of GFP-encoding LVs favored malignant cell survival and allowed to reveal the mechanism likely explaining the occurrence of transduced malignant cells in the clinic. This might be the reason for the surprisingly high transduction levels on malignant B cells in Figure 4.

Next, we aimed to evaluate our findings under conditions that more closely resemble clinical manufacturing conditions for which usually a lower LV dose is applied. Therefore, the transduction assay was repeated with B-ALL cells of one patient in a LV titration experiment with MOIs ranging from 40 to 1. Again, the LV stocks were titrated before by transduction of SupT1 cells with serially diluted LV and tested on activated T cells in advance to confirm the correct LV dose (Figure S8B). This time, an additional washing step was added to ensure sufficient removal of unbound LV. The transduction efficiency with anti-CD19 CAR-displaying LVs was enhanced by 3- to 5-fold compared to LVs displaying anti-CD20, anti-CD318, or no CAR on the surface even under low-dose conditions (MOIs 1 and 5) (Figure 5A). Interestingly, the overall transduction efficiency was decreased compared to the experiment in Figure 4, which may have been a result of additional washing. The transduction efficiency was analyzed on day 4 and in addition on 10 days post transduction to confirm stable gene transfer and the presence of functional LVs (Figure 5B). B-ALL cells are difficult to cultivate *in vitro* for a prolonged time.[15,17,20] Also here, the number of viable B-ALL cells decreased over the cultivation period of 10 days. But the ratio of GFP-expressing cells remained constant, with a distinct population of cells expressing GFP at high levels confirming stable gene transfer. Clinical manufacturing of CAR T cells requires the activation of T cells, which was omitted for the co-culture transduction experiments described in Figures 4 and 5. To evaluate the relevance of conditions resembling clinical manufacturing even more closely, the experiment was repeated with activated T cells instead of non-activated PBMCs (MOI 5) (Figure S9). The co-culture ratio was changed to 30% T cells and 70% malignant B cells, as typically seen for clinical manufacturing in patients with high blast counts. Cultivation and transduction conditions remained unchanged. The transduction efficiency of malignant B cells was increased 2-fold when using anti-CD19 CAR displaying LVs compared to LVs displaying anti-CD20, anti-CD318, or no CAR. Importantly, the transduction efficiency on T cells was 30%, which is comparable to results obtained for clinical manufacturing.[24] Thus, the described mechanism holds true under GMP-like production conditions. Of note, the transduction efficiency of malignant B cells was comparable to the results presented in Figure 5 (MOI 5), suggesting that even in the presence of activated T cells, malignant cells were transduced.

In summary, our data show that CAR-mediated binding of LVs to malignant B cells enhances the transduction efficiency of malignant B cells. This was observed for all applied LV doses in a dose-independent manner. Moreover, stable transgene expression was confirmed, excluding protein transfer by pseudotransduction.

Our results emphasize the need to prevent unintentional transduction of malignant cells. Thus, two potential approaches, blocking the

Figure 4. CD19 CARs displayed on LVs increase transduction efficiency on malignant cells

(A) A schematic representation of the experimental setup is shown. 30% malignant B-precursor cells of three different B-ALL patients were mixed to 70% PBMCs of a healthy donor. LV (MOI 10) and the LV-inhibitor raltegravir (RG) were added to respective samples. GFP-encoding LVs displaying different CAR proteins and LNGFR were added for 90 min at 37°C. Unbound LV was removed by three successive washing steps, and the transduction efficiency was analyzed 4 days post transduction by flow cytometry. (B) Gating strategy to identify malignant B cells co-cultured with PBMCs of the healthy donor (representative data for one donor). As control, samples containing either PBMCs or malignant B cells alone were analyzed by flow cytometry. Importantly, non-malignant B cells were identified by gating on the CD20+, CD19+ double-positive fraction, while malignant B cells were CD19+, CD20−. (C) Transduction of malignant B cells co-cultured with PBMCs is shown (representative data for one donor). Respective samples were treated with raltegravir (w/ RG) or left untreated (w/o RG). (D) Average data of triplicates of two patients (pink, turquoise) and a single measurement for one patient (black) ± SD are shown. **p = 0.0082, paired two-tailed t test. ns, not significant (p = 0.0772 [CD20-CAR/w/o], p = 0.1414 [CD318-CAR/w/o]); paired two-tailed t test.

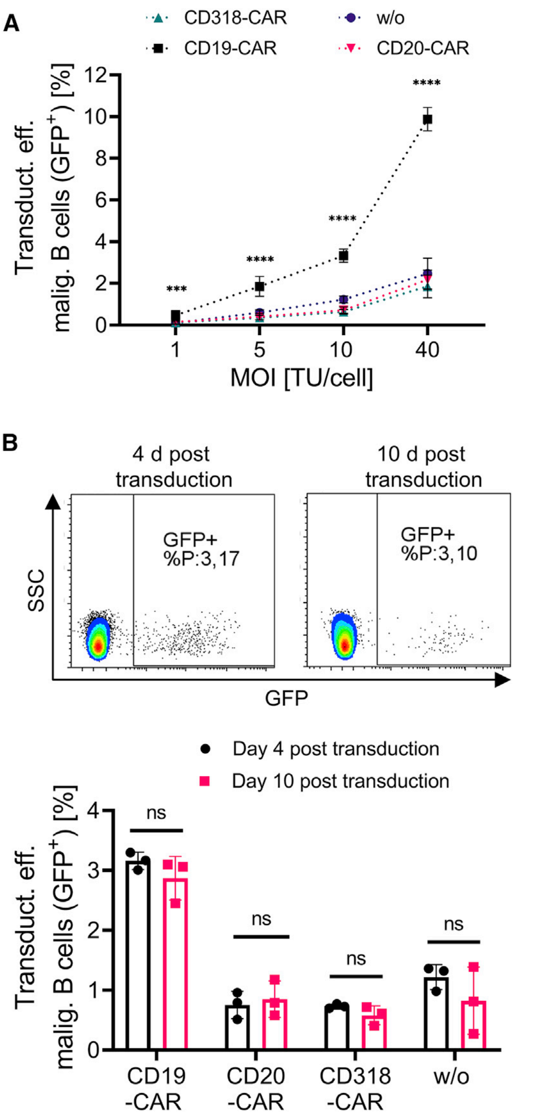

Figure 5. Dose-dependent transduction of malignant cells and long-term culture

(A) A co-culture of malignant B-precursor cells of one B-ALL patient and PBMCs of a healthy donor were transduced with GFP-encoding LVs displaying different CAR proteins and LNGFR in doses ranging from MOI 1 to 40. Average data of 6 samples from two independent experiments ± SD are shown. ****p < 0.0001, ***p = 0.007 (CD20-CAR/CD19-CAR), 0.003 (CD318-CAR/CD19-CAR), 0.002 (w/o/CD19-CAR), ordinary one-way ANOVA. (B) The samples transduced with an LV dose of MOI 10 were analyzed 4 days and 10 days post transduction to confirm stable transgene expression. Average data of 3 samples from one experiment ± SD is shown. ns, not significant (p = 0.668 [CD19-CAR], 0.9908 [CD20-CAR], 0.95 [CD318-CAR], 0.387 [w/o]); ordinary two-way ANOVA.

CAR-antigen by adding antibodies during the transduction and a controllable adapter-mediated CAR (Adapter-CAR) approach, were evaluated. For the first approach the co-culture transduction experiment was repeated in presence of a CD19-blocking antibody (Figure S9). Compared to the transduction in absence of the blocking antibody, the transduction efficiency with anti-CD19 CAR-displaying LVs was decreased by 50%, while the transduction with LVs displaying control or no CARs remained constant at high background levels. For the second approach, transduction of malignant cells with LVs displaying Adapter-CAR—a CAR construct that requires a biotinylated adapter molecule to bind to the target—was evaluated (Figure S10).[25] As expected, Adapter–CAR displayed on the surface of LVs did not induce significantly higher transduction efficiency levels on malignant B cells compared to transduction with control LVs displaying no CAR. Of note, both strategies did not prevent VSV-G LV binding and subsequent transduction of the malignant cells completely, which is reflected in background transduction levels of 1% for both conditions.

DISCUSSION

We describe here for the first time that CARs are displayed on LVs, mediating binding of LVs to CAR antigen-expressing cells. Subsequently, higher transduction efficiencies on leukemic B cells derived from three B-ALL patients were detected with LVs displaying anti-CD19 CARs.

Although the transfer of specific proteins from producer cells to HIV particles and lentiviral vectors is well documented, to our knowledge the display of CAR protein in LVs and their impact has not been shown before. Jamali and colleagues[9] could detect low levels of CAR protein within their LV preparation, but evidence of CAR display by lentiviral particles was not provided. The level of incorporation of a host cell protein into the LV envelope occurs in a non-random manner.[8] Data support a major role of the cytoplasmic domain of gp41 for glycoprotein incorporation, underlining the importance of intracellular domains.[26] For instance, engineering LV envelope proteins to restrict and/or expand the tropism typically require modifications on the cytoplasmatic domain to enable pseudotyping.[27] The cytoplasmatic tail of the CAR in our studies consists of 4-1BB- and CD3z-signaling domains, which were not expected to support efficient incorporation into the viral envelope as suggested by Jamali et al.[9] Here, we evaluated CAR display on VSV-G pseudotyped LVs containing signaling domains that were successfully applied in the clinic. However, alternative CAR designs and alternative vector systems may behave differently and could be investigated in future studies.

To exclude that the targeted epitope may influence particle binding, in total three different B cell-specific CARs were evaluated for binding to B cells. All CAR-displaying particles could bind the target-expressing cells, however, at different frequencies. A multitude of factors can influence the target-binding efficiency, such as affinity of the scFv for its target, the antigen expression level on the target cell, the stability of the CAR, or even the proximal or distal position of the epitope targeted. In particular, antigen density could influence the avidity and binding of the particles and should be considered when comparing CD19- and CD20-specific binding, as expression levels vary[28]. Differences in stability or expression levels could also influence the CAR protein level displayed on the LV, potentially contributing to

differential binding efficiency levels. Currently, reliable means to quantify displayed protein levels on LVs are not well established. Thus, further studies are needed to identify the factors determining the efficiency of CAR-mediated binding and entry of LV particles. Consequently, it may be advisable to ensure that the risks associated with a potential CAR binder in respect to CAR-mediated LV transduction is low, through the use of the assays described in this manuscript, in case the target cells are present during the transduction.

Two main strategies are commonly used to investigate pseudotransduction: long-term cultivation and transduction in presence of LV inhibitors.[29] In this study, we could confirm lentiviral gene transfer by long-term cultivation of primary B-ALL cells over 10 days. In addition, in every transduction assay, transgene expression was sensitive toward treatment with the LV inhibitor raltegravir, further confirming stable lentiviral gene transfer and excluding pseudotransduction. Furthermore, pseudotransduction by e.g. exosomes typically results in a heterogenous, smear type of expression without clear population, while LVs typically induce the generation of a distinct, bright population as presented here.

In line with previous studies, we could show efficient transduction of malignant B cells with VSV-G LVs.[17–20] Of note, in contrast to RVs, which require the breakdown of the nuclear membrane during mitosis, lentiviral vectors are known to allow transduction of resting cells.[30–32] Since the main receptor of VSV-G (LDLR) was not expressed by the malignant cells, analysis of expression of alternative receptors or alterations in viral defense mechanism (such as intracellular restriction factors of lentiviral transduction previously identified, e.g., IFITM3, SAMHD1, and TRIM[33–36]) could help explain the increased transduction of malignant versus healthy B cells.

Compared to the conditions applied for clinical manufacturing, the cultivation and transduction conditions of this study were adapted in various aspects to enable better detection of this rare transduction event. First, cytokines and media supplements favored B-ALL cell survival to enable prolonged cultivation *in vitro* and analysis of transgene expression levels upon transduction. Second, T cell activation reagents were not applied to better cultivate and analyze the B-ALL cells (Figures 4 and 5). Consequently, the VSV-G receptor was not expressed at high levels, the transduction efficiency on T cells remained low, and the LV dose was not reduced by cellular uptake on T cells. Third, application of GFP-encoding LV inhibited generation of CAR T cells and thereby CAR-mediated lysis of non-transduced B-ALL cells. Fourth, research-grade self-made GFP-encoding LVs were applied, which differ in quality from GMP-grade LVs. In summary, the transduction efficiency levels of B-ALL cells under clinical conditions are most likely reduced compared to the levels observed in this study. This may explain the discrepancy between the relatively high transduction efficiency levels observed here and the presence of only one report describing this type of resistance. Of note, we could also show enhanced transduction of malignant B cells with anti-CD19 CAR-displaying LVs even in the presence of activated T cells. Data from more patients would be helpful to better quantify the overall frequency of this rare type of relapse and the relevance of the mechanism discovered in this study. In particular, it remains unknown which CAR expression levels are required on B-ALL cells to induce sufficient masking of CD19 antigen. Presumably, not every transduction event results in CAR expression levels that inhibit CAR T cell function.

Based on our data, we postulate that the transduction of malignant cells is a two-step process. LVs bind specifically to malignant cells via the displayed CAR, then the cells are subsequently transduced with cell-bound LVs by the activity of VSV-G fusing the viral and cellular membrane. Consequently, the described mechanism also depends on the level of expression of LDLR and its family members on B-ALL cells, which could vary from patient to patient. In case the natural receptors of VSV-G are expressed at low levels, the effect of CAR-surface display would be enhanced, promoting higher transduction efficiency of malignant cells. In contrast, when VSV-G receptors are expressed at higher levels, the effect of CAR-mediated binding could become less dominant. Further analysis, however, is needed to dissect the role of LDLR expression, VSV-G, and CAR in more detail. Moreover, the incorporation and display of CAR protein may be a general mechanism not only limited to the specific CARs evaluated here. This should be considered also for other CARs and antigen-expressing tumor cells potentially being present during the transduction.

Recently, a strategy to remove anti-CD19 CAR-expressing leukemic cells was presented using idiotype-specific CAR T cells specifically targeting anti-CD19 CAR-expressing cells.[37] However, this strategy targets not only the malignant CAR-expressing B cells but also the therapeutically active CAR T cells. Moreover, an additional CAR T cell product is required to eradicate the malignant CAR-expressing B cells. Here, we could show that blocking the CAR antigen by adding antibodies during the transduction and controllable adapter-mediated CAR approaches offers the possibility to reduce CAR-mediated LV binding during the transduction step. In the context of CAR T cells, blocking CARs with antibodies has been shown to be challenging (e.g., only a modest reduction in function of anti-CD20 CAR T cells in presence of rituximab was detected).[38] Analogously, protocol optimization and careful CAR-specific evaluation is most likely required, if antigen blocking is used to abolish malignant cell transduction. Certainly, antigen blocking or using adapter-mediated CAR approaches are only able to reduce the VSV-G-mediated transduction of malignant cells, which makes additional methods to block B cell transduction necessary.

By focusing on potential risk factors at the transduction step, T cell enrichment and/or tumor cell depletion from the starting material are efficient strategies creating a defined T cell product containing fewer residual malignant B cells. In addition, our study highlights the need for more selective vector systems providing an additional layer of safety.

MATERIALS AND METHODS

LV generation and titration

VSV-G pseudotyped LVs were produced as described before by transient transfection of HEK293T cells.[27] Alternatively, HEK293T cells

stably expressing CARs and LNGFR were used to generate GFP-encoding LV displaying CAR/LNGFR. The LV was harvested 48 h post transfection. To remove cellular debris, the supernatant was collected and centrifuged for 10 min at 1,000 rpm, followed by filtration through a 0.45 μm filter. To concentrate, the filtered supernatant was centrifuged for 24 h at 4°C with 5,350 × *g* through a 20% sucrose cushion. The pelleted LV was resuspended in precooled PBS, aliquoted, and stored at −80°C for later use. Transfer plasmids encoding GFP under a spleen focus forming virus (SFFV) promotor or a polycistronic expression cassette containing second-generation CARs under phosphoglycerat kinase-1 promotor (PGK) promotor were used with 4-1BB/CD3 zeta stimulatory domain and a CD8- or IgG4-spacer followed by a P2A element-linked truncated LNGFR. The anti-CD20 CAR construct comprised a Leu16-derived scFv with leading heavy chain, while the anti-CD19 CAR construct was comprised of an FMC63-derived scFV with leading light chain.[39,40] The ofatumumab-derived CAR was generated by Gibson cloning, inserting the scFV sequence with leading heavy chain into the anti-CD20 CAR construct (https://go.drugbank.com/drugs/DB06650). A third-generation adapter-CAR construct containing CD28 and 41BB co-stimulatory domains was used.[25] LV titers were determined by transducing SupT1 cells with serially diluted GFP- or CAR-encoding LV in RPMI (BioWest, Nuaillé, France) supplemented with 2 mM stable glutamine (Lonza, Basel, Switzerland). 96 h post transduction, the transduction efficiency was determined by flow cytometry determining the ratio of GFP- or LNGFR-positive cells (clone: REA844, Miltenyi Biotec, Bergisch Gladbach, Germany). The ratio of GFP- or LNGFR-positive cells, the dilution factor, and the volume of lentiviral vector particles applied were used to calculate the LV titer (i.e., transducing units per volume [TU/mL]).

Generation of HEK293T cells stably expressing CAR and LNGFR

3.5×10^5 HEK293T cells were seeded in cultivation medium (DMEM [BioWest, Nuaillé, France]/10% fetal calf serum [FCS, Biochrom, Berlin, Germany]) in 12-well plates. 24 h post seeding, the medium was removed and VSV-G-pseudotyped LVs encoding for anti-CD19 CAR, anti-CD20 CAR, or anti-CD318 CAR and LNGFR were added in DMEM without (w/o) FCS at an MOI of 30 to the cells. 24 h post transduction, the medium containing excess LV was removed and 2 mL fresh cultivation medium was added. LNGFR expression of the cells was analyzed 1 week post transduction by flow cytometry (clone: REA844, Miltenyi Biotec, Bergisch Gladbach, Germany). The bulk population was used 2 weeks post transduction for generation of LV.

CAR display and PBMC binding

For detection of CAR proteins displayed on LVs, 2×10^5 SupT1 cells were incubated for 1 h at 4°C with LVs (MOI = 40) in RPMI medium w/o supplements in 96 well-round bottom plates. Subsequently, the supernatant was removed, and cells were washed once with cold PBS/EDTA/BSA. Cells with bound LV were stained for LNGFR, VSV-G (clone: 8G5F11, Kerafast, Boston, MA, USA), or with protein L (Genscript, NJ, USA)[41] to detect the CAR scFV and fixed using 2% paraformaldehyde in PBS/EDTA/BSA. For the identification of the bound cell type, 2.5×10^5 freshly isolated PBMCs were seeded in TexMACS medium (w/o supplements) (Miltenyi Biotec, Bergisch Gladbach, Germany) and incubated for 1 h at 4°C with LV (MOI = 40). The cells were washed after LV incubation as described before with cold PBS/EDTA/BSA and stained for viability (7AAD, Miltenyi Biotec, Bergisch Gladbach, Germany) and surface expression of CD3, CD14, CD16, CD56, CD19, and CD20 (clone: REA613, REA599, REA423, REA196, REA675, REA780, and REA844, Miltenyi Biotec, Bergisch Gladbach, Germany). The cells were fixed using 2% paraformaldehyde in PBS/EDTA/BSA. For blocking of the CD19-antigen, unstimulated PBMC of three donors were preincubated with increasing concentrations of a CD19-specific antibody (clone: LT-19, Miltenyi Biotec, Bergisch Gladbach, Germany) ranging from 0–5,000 ng/mL for 30 min at 4°C followed by incubation with LVs as described above. Flow cytometry was performed using the MACSQuant Analyzer 10 or MACSQuant X (Miltenyi Biotec, Bergisch-Gladbach, Germany), and the data were analyzed using FlowLogic (Inivai Technologies, Mentone Victoria, Australia).

Malignant B cells

Leukemic cells of B-ALL patients were isolated from the negative fraction of leukapheresis products post T cell enrichment during CAR-T cell production within a CAR-T clinical study (ClinicalTrials.gov: NCT03853616) using Ficoll (PAN Biotech, Aidenbach, Germany) gradient centrifugation or from bone marrow aspirates at initial diagnosis. The study was approved by the University of Muenster Ethical Board, and informed consent was obtained from donors, patients, and/or their legal guardians in accordance with the Declaration of Helsinki.

Co-culture transduction

1.75×10^5 freshly isolated PBMCs of healthy donors were added to 0.75×10^5 malignant B cells and were seeded in RPMI, 5% stable glutamine, SCF/IL-3/Flt-3 (Miltenyi Biotec, Bergisch Gladbach Germany) in 96-well round-bottom plates. As control, samples were preincubated for 30 min with raltegravir (1 μM) (Sigma Aldrich, St. Louis, MO, USA) and GFP-encoding LV displaying anti-CD19 CAR, anti-CD20 CAR, anti-CD318 CAR, or no CAR applying the indicated MOI for 1.5 h at 37°C. For blocking of the CD19 antigen, a biotinylated CD19-specific antibody (clone: LT19, Miltenyi Biotec, Bergisch Gladbach Germany) was added at a concentration of 2.5 μg/mL to the cells, followed by 30 min incubation at 4°C prior to transduction. Excess LV was removed by three successive washing steps using RPMI w/o supplements. Cells were cultivated in RPMI (20% FCS, 2 mM stable glutamine, 300 ng/mL SCF/60 ng/mL IL-3/300 ng/mL Flt-3 [Miltenyi Biotec, Bergisch Gladbach, Germany] ± Raltegravir). 4 days post transduction, the cells were stained for viability and surface expression of CD45 (clone: REA747, Bergisch Gladbach, Germany), CD3, CD14, CD16, CD56, CD19, and CD20. Transduction efficiency was analyzed by quantification of GFP-positive cells. Flow cytometry was performed using the MACSQuant Analyzer 10 (Miltenyi Biotec, Bergisch-Gladbach, Germany), and the data were analyzed with FlowLogic (Inivai Technologies, Mentone, VIC, Australia).

LV immobilization assay

In this assay, LVs were specifically immobilized via the CAR antigen in an ELISA plate and then overlaid with SupT1 cells to determine the presence of functional LVs by measuring the transduction efficiency after 8 days. For the immobilization of anti-CD19 CAR-displaying LVs, the anti-CD19 CAR detection reagent (Miltenyi Biotec, Bergisch Gladbach) consisting of a recombinantly expressed, biotinylated extracellular domain of CD19 containing the epitope of FMC63 was used. Anti-CD20 CAR-displaying LVs were immobilized via a recombinant CD20 peptide.

For this, a 96-well ELISA plate was either coated for 1 h at room temperature (RT) with 100 μL of streptavidin (1 μg/mL) or was left untreated followed by incubation with 300 μL of PBS-Tween/BSA (2%) for 1 h at RT. Next, biotinylated CD19-CAR detection reagent and the recombinant CD20 peptide was diluted 1:10 in PBS/BSA (2%). 100 μL of the diluted CD19-detection reagent was added to the wells with coated streptavidin, while 100 μL of the CD20-peptide was directly immobilized in the wells of an ELISA plate without streptavidin. Excess protein was removed by three successive washing steps with 300 μL PBS. Subsequently, CAR-displaying LVs (GFP-encoding) diluted in PBS/BSA (2%) were added to the wells, followed by an incubation for 1 h at RT to enable LV binding to the CAR antigen. Unbound LVs were removed by four successive washing steps using 300 μL of PBS. Next, 2×10^5 SupT1 cells were added in 200 μL RPMI medium (5 mM stable glutamine, 5% penicillin/streptomycin). The following day, 90 μL fresh RPMI (5 mM stable glutamine, 10% FCS) was added to enable culture until the end of the experiment when gene transfer is complete and steady-state expression levels of the transgene are reached. The transduction efficiency was analyzed 8 days post transduction by quantification of GFP-positive cells among viable cells using flow cytometry.

SUPPLEMENTAL INFORMATION

Supplemental information can be found online at https://doi.org/10.1016/j.omtm.2021.02.013.

ACKNOWLEDGMENTS

We would like to thank Jens Hellmer for providing technical expertise for LC-MS/MS analysis. This project has received funding from the European Union's Horizon 2020 Research and Innovation Program under grant agreement no. 667980 (CARAT) to N.C., C.K., T.C., T.S., and A.D.K.

AUTHOR CONTRIBUTIONS

N.C. and C.K. performed the experiments. D.L. assisted in performing the experiments. D.A. performed the LC-MS/MS analysis. T.H., I.B., F.B., S.K., C.R., and T.F. provided patient samples. B.K., T.C., and M.A. provided critical discussion. D.S. provided the anti-CD318 CAR construct. N.C., T.S., and A.D.K. formulated the ideas, designed the experiments, and wrote the manuscript.

DECLARATION OF INTERESTS

N.C., C.K., D.L., T.H., D.S., B.K., S.K., I.B., M.A., T.S., and A.D.K. are employees of Miltenyi Biotec B.V. & Co. KG. N.C., T.S., and A.D.K. have relevant IP to the findings disclosed. No competing financial interests exist for the remaining authors.

REFERENCES

1. Shah, N.N., and Fry, T.J. (2019). Mechanisms of resistance to CAR T cell therapy. Nat. Rev. Clin. Oncol. *16*, 372–385.
2. Ruella, M., Xu, J., Barrett, D.M., Fraietta, J.A., Reich, T.J., Ambrose, D.E., Klichinsky, M., Shestova, O., Patel, P.R., Kulikovskaya, I., et al. (2018). Induction of resistance to chimeric antigen receptor T cell therapy by transduction of a single leukemic B cell. Nat. Med. *24*, 1499–1503.
3. Maude, S.L., Laetsch, T.W., Buechner, J., Rives, S., Boyer, M., Bittencourt, H., Bader, P., Verneris, M.R., Stefanski, H.E., Myers, G.D., et al. (2018). Tisagenlecleucel in children and young adults with B-cell lymphoblastic leukemia. N. Engl. J. Med. *378*, 439–448.
4. Maude, S.L., Frey, N., Shaw, P.A., Aplenc, R., Barrett, D.M., Bunin, N.J., Chew, A., Gonzalez, V.E., Zheng, Z., Lacey, S.F., et al. (2014). Chimeric antigen receptor T cells for sustained remissions in leukemia. N. Engl. J. Med. *371*, 1507–1517.
5. Johnson, S., Wheeler, J.X., Thorpe, R., Collins, M., Takeuchi, Y., and Zhao, Y. (2018). Mass spectrometry analysis reveals differences in the host cell protein species found in pseudotyped lentiviral vectors. Biologicals *52*, 59–66.
6. Segura, M.M., Garnier, A., Di Falco, M.R., Whissell, G., Meneses-Acosta, A., Arcand, N., and Kamen, A. (2008). Identification of host proteins associated with retroviral vector particles by proteomic analysis of highly purified vector preparations. J. Virol. *82*, 1107–1117.
7. Tremblay, M.J., Fortin, J.F., and Cantin, R. (1998). The acquisition of host-encoded proteins by nascent HIV-1. Immunol. Today *19*, 346–351.
8. Burnie, J., and Guzzo, C. (2019). The Incorporation of Host Proteins into the External HIV-1 Envelope. Viruses *11*, 85.
9. Jamali, A., Kapitza, L., Schaser, T., Johnston, I.C.D., Buchholz, C.J., and Hartmann, J. (2019). Highly Efficient and Selective CAR-Gene Transfer Using CD4- and CD8-Targeted Lentiviral Vectors. Mol. Ther. Methods Clin. Dev. *13*, 371–379.
10. Ziegler, L., Yang, L., Joo, Ki., Yang, H., Baltimore, D., and Wang, P. (2008). Targeting lentiviral vectors to antigen-specific immunoglobulins. Hum. Gene Ther. *19*, 861–872.
11. Chan, L., Nesbeth, D., Mackey, T., Galea-Lauri, J., Gäken, J., Martin, F., Collins, M., Mufti, G., Farzaneh, F., and Darling, D. (2005). Conjugation of lentivirus to paramagnetic particles via nonviral proteins allows efficient concentration and infection of primary acute myeloid leukemia cells. J. Virol. *79*, 13190–13194.
12. Finkelshtein, D., Werman, A., Novick, D., Barak, S., and Rubinstein, M. (2013). LDL receptor and its family members serve as the cellular receptors for vesicular stomatitis virus. Proc. Natl. Acad. Sci. USA *110*, 7306–7311.
13. Amirache, F., Lévy, C., Costa, C., Mangeot, P.-E., Torbett, B.E., Wang, C.X., Nègre, D., Cosset, F.-L., and Verhoeyen, E. (2014). Mystery solved: VSV-G-LVs do not allow efficient gene transfer into unstimulated T cells, B cells, and HSCs because they lack the LDL receptor. Blood *123*, 1422–1424.
14. Nash, K.L., and Lever, A.M.L. (2004). Green fluorescent protein: green cells do not always indicate gene expression. Gene Ther. *11*, 882–883.
15. Bruserud, Ø., Glenjen, N., Ryningen, A., and Ulvestad, E. (2003). In vitro culture of human acute lymphoblastic leukemia (ALL) cells in serum-free media; a comparison of native ALL blasts, ALL cell lines and virus-transformed B cell lines. Leuk. Res. *27*, 455–464.
16. Nijmeijer, B.A., Szuhai, K., Goselink, H.M., van Schie, M.L.J., van der Burg, M., de Jong, D., Marijt, E.W., Ottmann, O.G., Willemze, R., and Falkenburg, J.H.F. (2009). Long-term culture of primary human lymphoblastic leukemia cells in the absence of serum or hematopoietic growth factors. Exp. Hematol. *37*, 376–385.
17. Biagi, E., Bambacioni, F., Gaipa, G., Casati, C., Golay, J., Biondi, A., and Introna, M. (2001). Efficient lentiviral transduction of primary human acute myelogenous and lymphoblastic leukemia cells. Haematologica *86*, 13–16.
18. Bonamino, M., Serafini, M., D'Amico, G., Gaipa, G., Todisco, E., Bernasconi, S., Golay, J., Biondi, A., and Introna, M. (2004). Functional transfer of CD40L gene in human B-cell precursor ALL blasts by second-generation SIN lentivectors. Gene Ther. *11*, 85–93.

19. Stripecke, R., Cardoso, A.A., Pepper, K.A., Skelton, D.C., Yu, X.J., Mascarenhas, L., Weinberg, K.I., Nadler, L.M., and Kohn, D.B. (2000). Lentiviral vectors for efficient delivery of CD80 and granulocyte-macrophage- colony-stimulating factor in human acute lymphoblastic leukemia and acute myeloid leukemia cells to induce antileukemic immune responses. Blood *96*, 1317–1326.

20. Mascarenhas, L., Stripecke, R., Case, S.S., Xu, D., Weinberg, K.I., and Kohn, D.B. (1998). Gene delivery to human B-precursor acute lymphoblastic leukemia cells. Blood *92*, 3537–3545.

21. Frecha, C., Lévy, C., Cosset, F.L., and Verhoeyen, E. (2010). Advances in the field of lentivector-based transduction of T and B lymphocytes for gene therapy. Mol. Ther. *18*, 1748–1757.

22. Frecha, C., Costa, C., Lévy, C., Nègre, D., Russell, S.J., Maisner, A., Salles, G., Peng, K.W., Cosset, F.L., and Verhoeyen, E. (2009). Efficient and stable transduction of resting B lymphocytes and primary chronic lymphocyte leukemia cells using measles virus gp displaying lentiviral vectors. Blood *114*, 3173–3180.

23. Gallardo, H.F., Tan, C., Ory, D., and Sadelain, M. (1997). Recombinant retroviruses pseudotyped with the vesicular stomatitis virus G glycoprotein mediate both stable gene transfer and pseudotransduction in human peripheral blood lymphocytes. Blood *90*, 952–957.

24. Castella, M., Caballero-Baños, M., Ortiz-Maldonado, V., González-Navarro, E.A., Suñé, G., Antoñana-Vidósola, A., Boronat, A., Marzal, B., Millán, L., Martín-Antonio, B., et al. (2020). Point-of-care CAR T-cell production (ARI-0001) using a closed semi-automatic bioreactor: Experience from an academic phase i clinical trial. Front. Immunol. *11*, 482.

25. Grote, S., Mittelstaet, J., Baden, C., Chan, K.C.-H., Seitz, C., Schlegel, P., Kaiser, A., Handgretinger, R., and Schleicher, S. (2020). Adapter chimeric antigen receptor (AdCAR)-engineered NK-92 cells: an off-the-shelf cellular therapeutic for universal tumor targeting. OncoImmunology *9*, 1825177.

26. Checkley, M.A., Luttge, B.G., and Freed, E.O. (2011). HIV-1 envelope glycoprotein biosynthesis, trafficking, and incorporation. J. Mol. Biol. *410*, 582–608.

27. Funke, S., Maisner, A., Mühlebach, M.D., Koehl, U., Grez, M., Cattaneo, R., Cichutek, K., and Buchholz, C.J. (2008). Targeted cell entry of lentiviral vectors. Mol. Ther. *16*, 1427–1436.

28. Horna, P., Nowakowski, G., Endell, J., and Boxhammer, R. (2019). Comparative Assessment of Surface CD19 and CD20 Expression on B-Cell Lymphomas from Clinical Biopsies: Implications for Targeted Therapies. Blood *134*, 5345.

29. Frecha, C., Costa, C., Nègre, D., Gauthier, E., Russell, S.J., Cosset, F.L., and Verhoeyen, E. (2008). Stable transduction of quiescent T cells without induction of cycle progression by a novel lentiviral vector pseudotyped with measles virus glycoproteins. Blood *112*, 4843–4852.

30. Roe, T., Reynolds, T.C., Yu, G., and Brown, P.O. (1993). Integration of murine leukemia virus DNA depends on mitosis. EMBO J. *12*, 2099–2108.

31. Miller, D.G., Adam, M.A., and Miller, A.D. (1990). Gene transfer by retrovirus vectors occurs only in cells that are actively replicating at the time of infection. Mol. Cell. Biol. *10*, 4239–4242.

32. Vigna, E., and Naldini, L. (2000). Lentiviral vectors: excellent tools for experimental gene transfer and promising candidates for gene therapy. J. Gene Med. *2*, 308–316.

33. Kahle, T., Volkmann, B., Eissmann, K., Herrmann, A., Schmitt, S., Wittmann, S., Merkel, L., Reuter, N., Stamminger, T., and Gramberg, T. (2015). TRIM19/PML Restricts HIV Infection in a Cell Type-Dependent Manner. Viruses *8*, 2.

34. Gramberg, T., Kahle, T., Bloch, N., Wittmann, S., Müllers, E., Daddacha, W., Hofmann, H., Kim, B., Lindemann, D., and Landau, N.R. (2013). Restriction of diverse retroviruses by SAMHD1. Retrovirology *10*, 26.

35. Laguette, N., Sobhian, B., Casartelli, N., Ringeard, M., Chable-Bessia, C., Ségéral, E., Yatim, A., Emiliani, S., Schwartz, O., and Benkirane, M. (2011). SAMHD1 is the dendritic- and myeloid-cell-specific HIV-1 restriction factor counteracted by Vpx. Nature *474*, 654–657.

36. Jia, R., Pan, Q., Ding, S., Rong, L., Liu, S.-L., Geng, Y., Qiao, W., and Liang, C. (2012). The N-Terminal Region of IFITM3 Modulates Its Antiviral Activity by Regulating IFITM3 Cellular Localization. J. Virol. *86*, 13697–13707.

37. Ruella, M., Barrett, D.M., Shestova, O., Perazzelli, J., Posey, A.D., Hong, S.J., Kozlowski, M., Lacey, S.F., Melenhorst, J.J., June, C.H., et al. (2020). A cellular antidote to specifically deplete anti-CD19 chimeric antigen receptor positive cells. Blood *135*, 505–509.

38. Rufener, G.A., Press, O.W., Olsen, P., Lee, S.Y., Jensen, M.C., Gopal, A.K., Pender, B., Budde, L.E., Rossow, J.K., Green, D.J., et al. (2016). Preserved activity of CD20-Specific chimeric antigen receptor-expressing T cells in the presence of rituximab. Cancer Immunol. Res. *4*, 509–519.

39. Lock, D., Mockel-Tenbrinck, N., Drechsel, K., Barth, C., Mauer, D., Schaser, T., Kolbe, C., Al Rawashdeh, W., Brauner, J., Hardt, O., et al. (2017). Automated Manufacturing of Potent CD20-Directed Chimeric Antigen Receptor T Cells for Clinical Use. Hum. Gene Ther. *28*, 914–925.

40. Schneider, D., Xiong, Y., Wu, D., Nölle, V., Schmitz, S., Haso, W., Kaiser, A., Dropulic, B., and Orentas, R.J. (2017). A tandem CD19/CD20 CAR lentiviral vector drives on-target and off-target antigen modulation in leukemia cell lines. J. Immunother. Cancer *5*, 42.

41. Zheng, Z., Chinnasamy, N., and Morgan, R.A. (2012). Protein L: a novel reagent for the detection of chimeric antigen receptor (CAR) expression by flow cytometry. J. Transl. Med. *10*, 29.

OMTM, Volume *21*

Supplemental information

Anti-CD19 CARs displayed at the surface of lentiviral vector particles promote transduction of target-expressing cells

Nicole Cordes, Carolin Kolbe, Dominik Lock, Tatjana Holzer, Deborah Althoff, Daniel Schäfer, Franziska Blaeschke, Bettina Kotter, Sandra Karitzky, Claudia Rossig, Toni Cathomen, Tobias Feuchtinger, Iris Bürger, Mario Assenmacher, Thomas Schaser, and Andrew D. Kaiser

Supplemental Methods

Monoculture transduction

One day prior transduction 200 µl of B cells with a density of 1 x10^6 cells/ml were seeded in 200 µl StemMACS HSC Expansion Medium XF supplemented with 50 IU/ml IL-4, CD40-L with 3.6 µl/ml cross-linking antibody in 96 well flat bottom plates. Directly prior transduction 50 µl of the growth medium was removed by pipetting. The LV (40 TU/cell) diluted in 50 µl StemMACS HSC Expansion Medium XF (50 IU/ml IL-4)) was added to the cells. One day post transduction medium was exchanged with 200 µl StemMACS HSC Expansion Medium XF supplemented with 50 IU/ml IL-4, CD40-L with 3.6 µl/ml crosslinking antibody and 5 % AB serum. Transduction efficiency was analyzed 14 days post transduction.

Malignant B cells alone were seeded in a density of 2.66x10^6 cell/ml in 150 µl RPMI (2 mM stable Glutamine, SCF/IL-3/Flt-3). Respective samples were preincubated for 30min with Raltegravir (1µM) and GFP-encoding LV was added (10 TU/cell). The medium of the malignant cells transduced in absence of PBMC was replaced 24 h post transduction with RPMI (20 % FCS, 2 mM stable Glutamine, SCF/IL-3/Flt-3, (Miltenyi Biotec, Bergisch Gladbach, Germany) +/- Raltegravir). 4 days post transduction the cells were stained for viability and surface expression of CD3, CD14, CD16, CD56, CD19, CD20. Transduction efficiency was analyzed by quantification of GFP positive cells. Flow cytometry was performed using the MACSQuant Analyzer 10 (Miltenyi Biotec, Bergisch-Gladbach, Germany) and the data was analyzed with FlowLogic (Inivai Technologies, Mentone Victoria, Australia).

Co-culture transduction with activated T cells

Primary T cells of two healthy donors were isolated one day prior to transduction using the PAN T cell isolation kit (Miltenyi Biotec, Bergisch Gladbach, Germany) and activated by cultivation over night in TexMACS medium supplemented with 12.5ng/ml IL7, 12.5ng/ml IL15 and Transact (Miltenyi Biotec, Bergisch Gladbach, Germany). A mixture of activated T cells of healthy two donors (30 %) and malignant B cells (70 %) were seeded in a density of 2.66x10^6 cell/ml in 150 µl RPMI (2 mM stable Glutamine, SCF/IL-3/Flt-3). Respective samples were preincubated for 30min with Raltegravir (1µM) and GFP-encoding LV displaying anti-CD19 CAR, anti-CD20 CAR, anti-CD318 CAR or no CAR (5 TU/cell) was added. Excess LV was removed by three successive washing steps after 1.5 h at 37°C incubation using RPMI w/o supplements for co-culture transductions. Cells were cultivated in RPMI (20 % FCS, 2 mM stable Glutamine, SCF/IL-3/Flt-3, (Miltenyi Biotec, Bergisch Gladbach, Germany) +/- Raltegravir). 4 days post transduction the cells were stained for viability and surface expression of CD45, CD3, CD14, CD16, CD56, CD19, CD20. Transduction efficiency was analyzed by quantification of GFP positive cells. Flow cytometry was performed using the MACSQuant Analyzer 10 (Miltenyi Biotec, Bergisch-Gladbach, Germany) and the data was analyzed with FlowLogic (Inivai Technologies, Mentone Victoria, Australia).

Production of GALV-RV

GALV pseudotyped RVs were produced by transient transfection of HEK293T cells or HEK-293T cells stably expressing CARs and LNGFR to generate GFP-encoding RV displaying CAR/LNGFR. For this 19.3µg pHIT, 25.7µg pLEGFP-N1 and 9.7µg of pCOLT-GALV. The RV was harvested 48 h post transfection. To remove cellular debris, the supernatant was collected, centrifuged for 10 min at 1000 rpm, followed by filtration through a 0.45 µm filter. To concentrate, the filtered supernatant was centrifuged for 24 h at 4 °C with 5350xg through a 20 % sucrose cushion. The pelleted RV was resuspended in precooled PBS, aliquoted and stored at -80 °C for later use. The transfer plasmids encoding a RV titers were determined by transducing HT1080 cells with serially diluted GFP encoding RV in DMEM (BioWest, Nuaillé, France) without supplements. 72 h post transduction the transduction efficiency was determined by flow cytometry determining the ratio of GFP. The ratio of GFP positive cells, the dilution factor and the volume of retroviral vector particles applied was used to calculate the RV titer (i.e. transducing units per volume (TU/ml)).

Binding Assay with GALV-RV

The protocol described under Material and Methods of the main manuscript was followed. A LV dose of 0.5TU/cell was applied.

In solution digest of lentiviral vector

Purified and concentrated GMP-grade lentiviral vector samples were heated up to 95 °C for 15 min in the presence of 0.05% Rapigest detergent (Waters, Eschborn, Ger). Samples were digested with trypsin overnight at 37 °C and subsequently acidified with 1 µl 10% trifluoracetic acid. C18 Stage tips (Thermo Scientific, Dreieich, Ger) were used to desalt and concentrate peptides. Peptides were eluted with 30 µl 80% acetonitrile and 0.1% formic acid, dried in a speed vac and dissolved in 20 µl 2% acetonitrile and 0.1% formic acid for analysis using mass spectrometry.

Lentiviral vector characterization using LC-MS/MS

LC-MS/MS was carried out using an Ultimate 3000 RSLCnano system coupled to a Thermo Scientific Orbitrap Fusion Lumos mass spectrometer (Thermo Fisher Scientific, Dreieich, Ger). The peptides were desalted in a trapping cartridge (Acclaim PepMap reversed phase C18, 5 µm,100Å, 100 µm x 2 cm length; Thermo Fisher Scientific) and separated on a C18 reversed phase nano-column (2 µm, 100 Å, 75 µm x 15 cm length; Thermo Fisher Scientific) using gradient conditions at a flow rate of 0.3 µl/min (mobile phase A: water with 0.1% formic acid; mobile phase B: acetonitrile with 0.1% formic acid). A step gradient with 2-30% B (5-40 min), 30-55% B (40-45 min) and 55-90% B (45-45.5 min was used with a total LC analysis time of 56 min. Separated and eluted peptides were sprayed at 2.2 kV needle voltage into the mass spectrometer. MS full scans were performed at 350-1500 m/z (mass-to charge-ratio) and a resolution of 120000 in the orbitrap. A top speed method was used with a intensity threshold of 5.0e4 for the peptides selected for MS/MS. The cycle time for each MS/MS was three sec and mass measurement was performed with the resolution of 15,000 in the orbitrap mass analyzer. Peptides were fragmented by collision induced dissociation at collision induced energy of 35% and dynamic exclusion was set to one. Mass spectra processing and database searching was carried out using PEAKS Studio 8.5 (Bioinformatics Solutions Inc., Waterloo, Canada). The precursor mass accuracy was set to 5 ppm and the fragment ion tolerance to 0.05 Da. Carbamidomethylation at cystein, pyro-glu from glutamine, oxidation of methionine, histidine and tryptophan as well as tryptophan oxidation to kynurenin were set as variable posttranslational modifications. For MS/MS peptide sequences up to three missed cleavages per peptide were allowed and the false discovery rate was set to 0.1%. A protein FASTA database including all lentiviral protein sequences of interest was used (Gag, Pol, VSV-G Env, LNGFR, CD19 CAR, CD20 CAR).

Supplemental Figures

Figure S1:

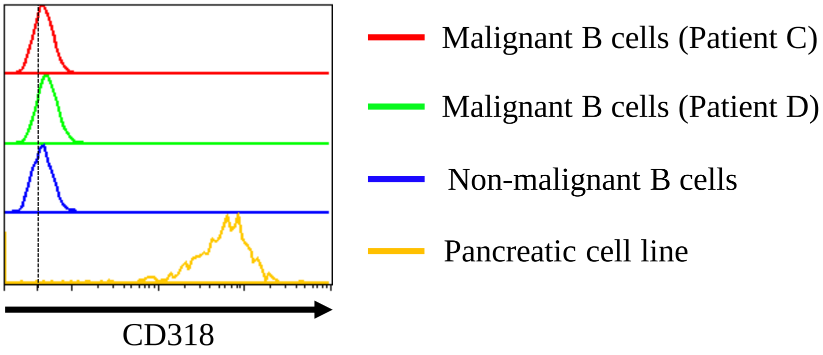

Figure S1. CD318 expression of healthy and malignant B cells.
Absence of expression of the CD318 on primary healthy B cells and primary B-ALL cells was confirmed by staining for CD318 and subsequent flow cytometry analysis. A CD318-expressing pancreatic cell line was used as positive control.

Figure S2:

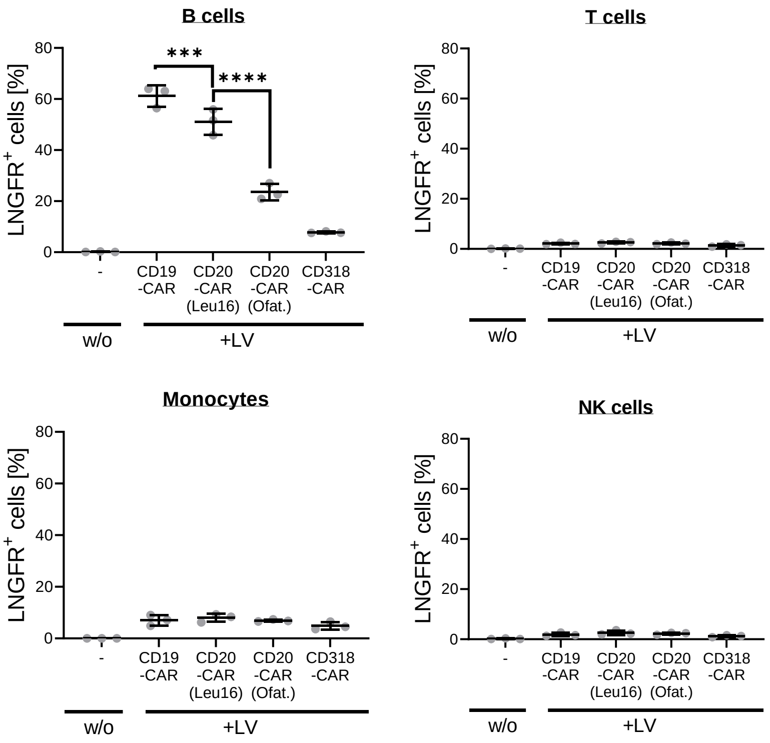

Figure S2. Binding of different CD20-CAR clones to unstimulated PBMC.
Unstimulated PBMC of three healthy donors were left untreated (-) or were incubated with anti-CD19 (clone: FMC63), anti-CD20 (clone:Leu16), anti-CD20 (clone: ofatumumab) or anti-CD318 CAR- and LNGFR-encoding LV (MOI= 10). After binding for 1 h at 4 °C, excess LV was removed by subsequent cell washing and cell-bound LV was detected by staining for LNGFR on the different cellular subsets namely T cells (CD3+, CD56-), Monocytes (CD3-, CD14+) and B cells (CD3-, CD19+). For each donor triplicates were analyzed. The average of triplicates for three donors ± SD is shown. ***P= .0006, ****P< .00001, 2-way ANOVA with multiple comparisons.

Figure S3:

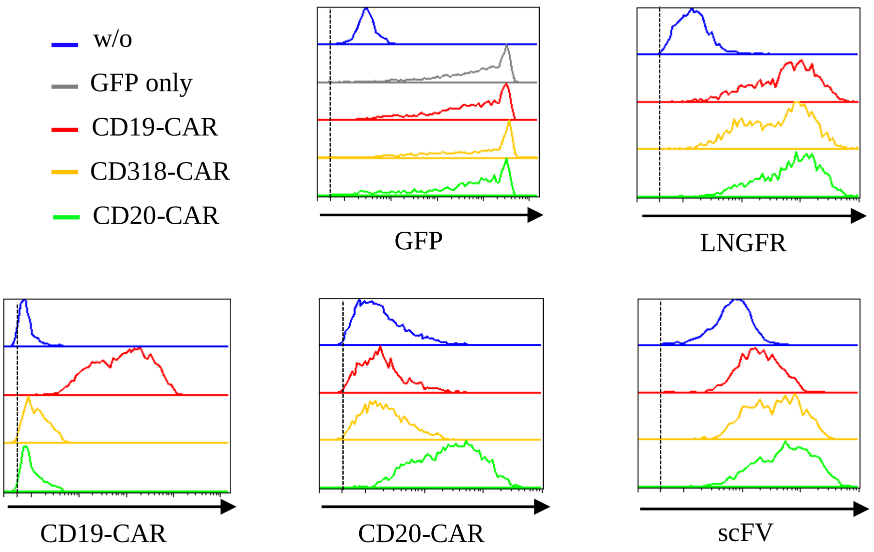

Figure S3. Generation of stably CAR expressing HEK293T cells.
HEK293T cells were genetically engineered by transduction with LVs to stably express an anti-CD19 CAR, anti-CD20 CAR or anti-CD318 CAR and LNGFR. Expression of the transgene was confirmed by staining for LNGFR, anti-CD19 CAR, anti-CD20 CAR or the murine scFV and subsequent flow cytometry analysis. The expression levels of LNGFR of the HEK293T cells expressing either the anti-CD19 CAR, the anti-CD20 CAR or the anti-CD318 CAR was compared to untreated HEK293T cells (w/o). The GFP expression during LV production was analyzed on the day of harvest and compared to HEK293T cells expressing no CAR (GFP only).

Figure S4:

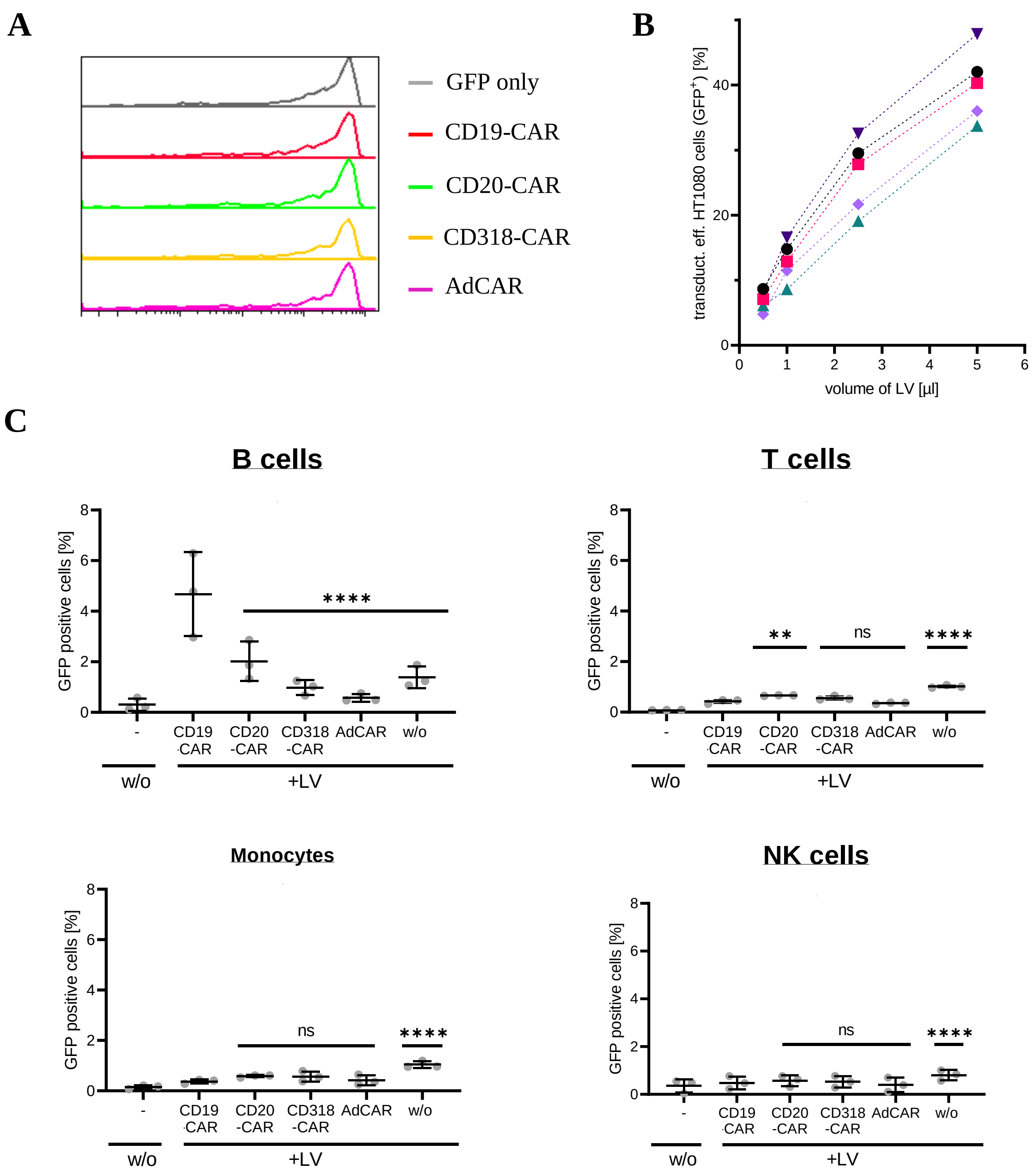

Figure S4. Binding of GALV-RV to unstimulated PBMC.

(**A**) The GFP expression during LV production was analyzed on the day of harvest and compared to HEK293T cells expressing no CAR (GFP only). (**B**) Titration of GALV-RV on HT1080 cells with serially diluted RV. (**C**) Unstimulated PBMC of three donors were left untreated (-) or were incubated with GFP-encoding RVs displaying anti-CD19, anti-CD20 or anti-CD318 CAR or AdCAR or no additional surface protein (w/o) (MOI= 0.5). After binding for 1 h at 4 °C, excess LV was removed by subsequent cell washing and cell-bound RV was detected by staining for LNGFR on the different cellular subsets namely T cells (CD3+, CD56-), Monocytes (CD3-, CD14+) and B cells (CD3-, CD19+). For each donor triplicates were analyzed. The average of triplicates for three donors ± SD is shown. Ns= not significant, ****P< .00001, 2-way ANOVA with multiple comparisons.

Figure S5:

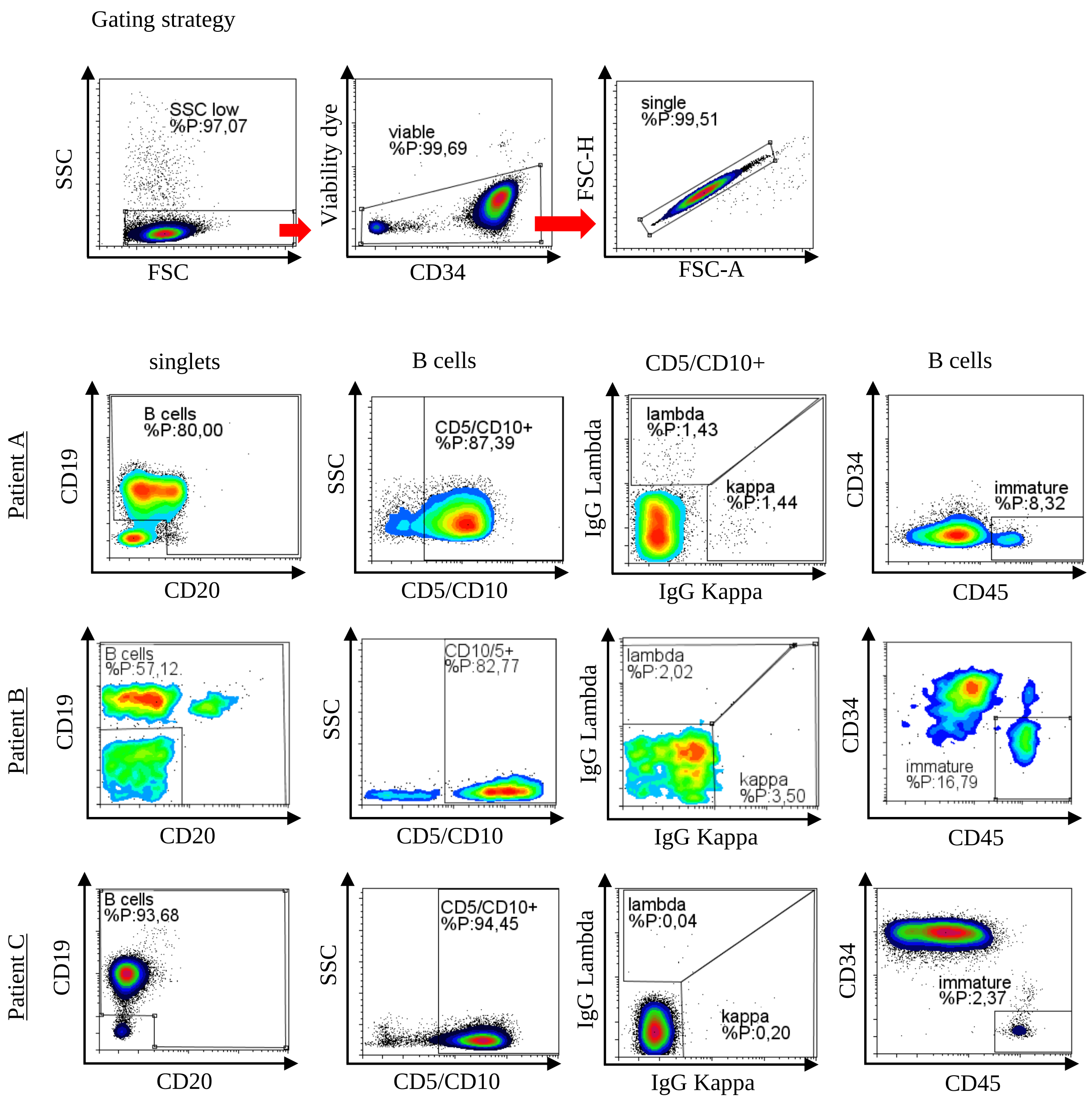

Figure S5. Characterization of primary malignant B cells.
Leukocytes were separated from residual erythrocytes/granulocytes by Ficoll (Pan-Biotech, cat# P04-601000). Obtained cells were stained for CD45, CD19, CD20, CD5, CD10, CD34, IgG Lambda and IgG Kappa and analyzed by flow cytometry. Results for three different B-ALL samples including gating strategy are shown.

Figure S6:

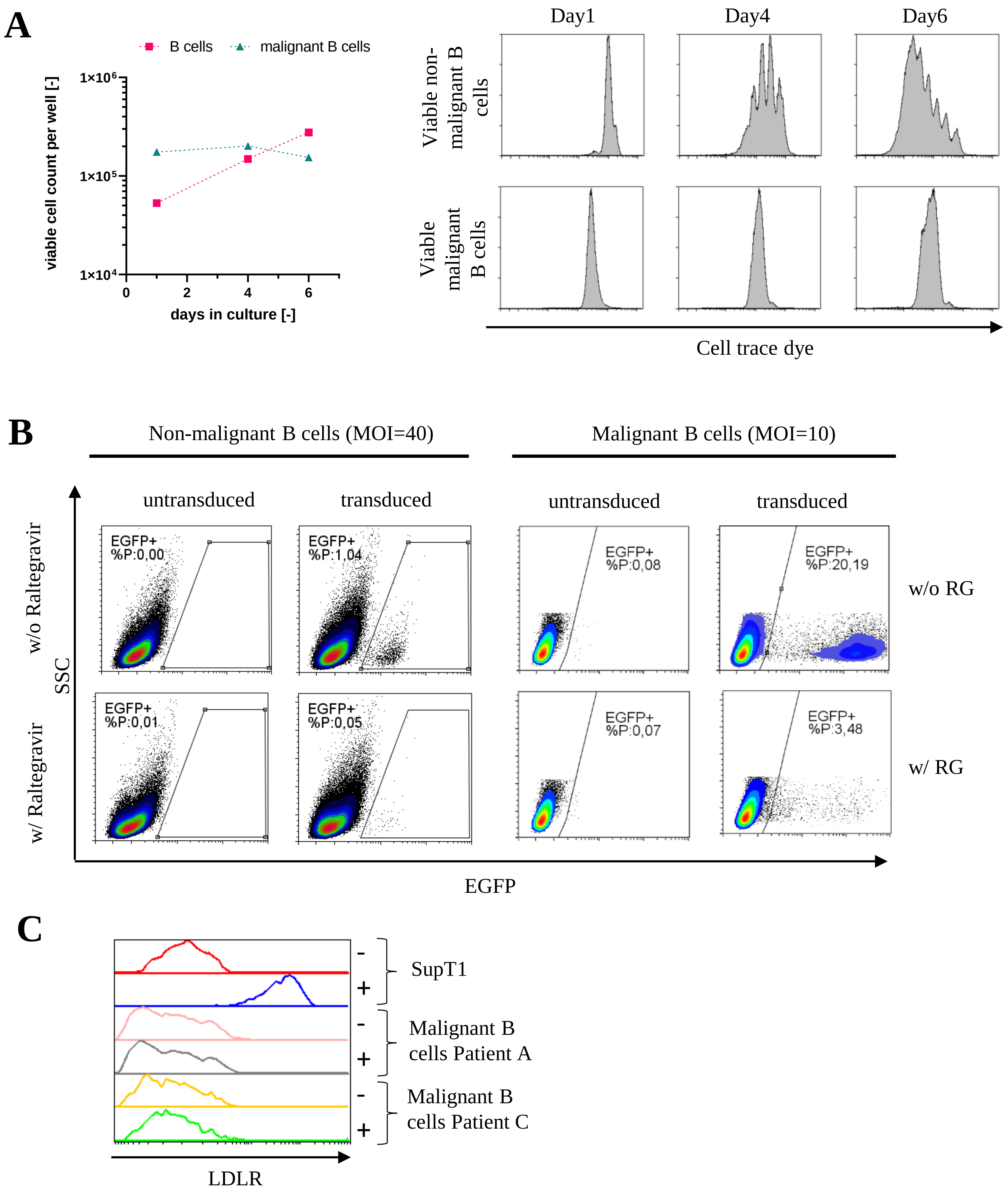

Figure S6. Comparison of transduction of healthy and malignant B cells.
(**A**) Cell proliferation of malignant and non-malignant B cells under chosen cultivation conditions evaluated by counting viable cells using the MACSQuant Analyzer 10 (left) and by following cell division using Violett Cell trace dye (Thermo Fisher Scientific, Waltham, Massachusetts, USA). (**B**) Healthy and malignant B cells were transduced with GFP-encoding LVs in presence (w/ RG) or absence (w/o RG) of the HIV integrase inhibitor Raltegravir. Transduction efficiency was analyzed by quantification of GFP^{+} cells 7 d (healthy B cells) or 5 d (malignant B cells) post transduction. (**C**) LDLR receptor staining on malignant B cells and on SupT1 cells (positive control) (clone: 472413, Thermo Fisher Scientific, Waltham, Massachusetts, USA) . Depicted are the results of staining without first antibody (-) as negative control and with both antibodies (+).

<u>Figure S7</u>:

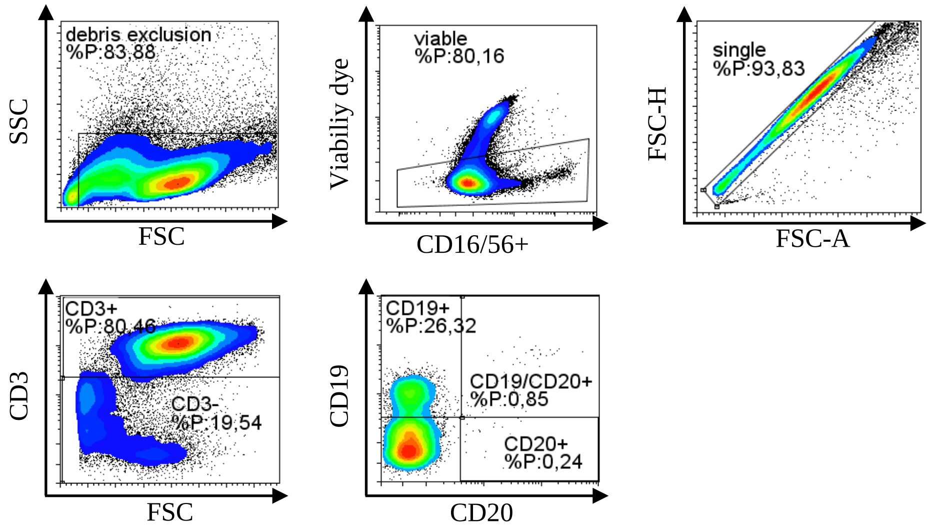

Figure S7. Gating strategy for analysis of transduction of primary malignant B cells.
The gating strategy for flow cytometry analysis of transduced malignant B cells in co-culture with healthy PBMC is shown. In a first step, cell debris was excluded. Next, viable and single cells were identified, and separated in CD3+ and CD3- cells. CD3- cells were separated in CD19+ and CD20+ populations. Transduction efficiency was analyzed on CD19+/CD20- cells.

Figure S8:

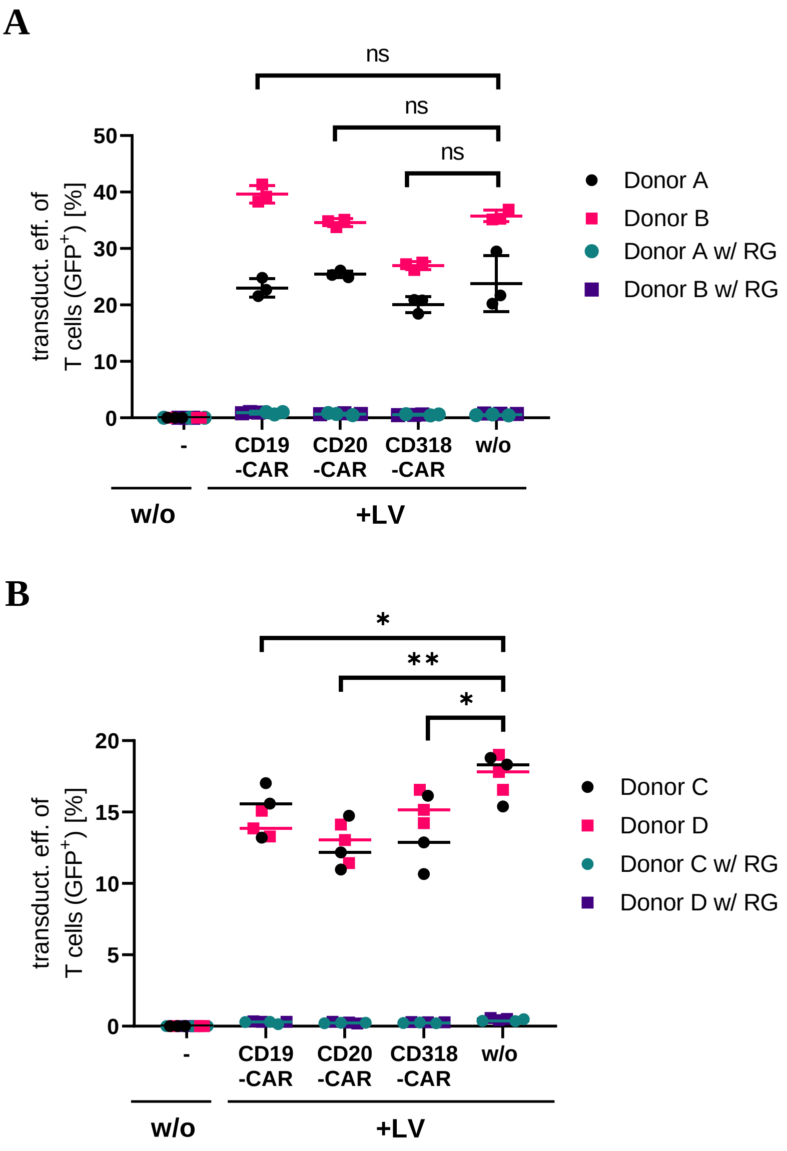

Figure S8. Transduction of activated T cells with GFP-encoding LVs displaying different CARs of different specificities and LNGFR.

Pan T cells were isolated from PBMC of two healthy donors using negative magnetic selection (Miltenyi Biotec, cat# 130-096-535). T cells were activated with Transact® (Miltenyi Biotec, cat# 130-111-160) overnight, respective samples were treated with the integrase inhibitor Raltegravir (RG) and transduced with GFP-encoding LVs displaying either the anti-CD19-, anti-CD20-, anti-CD318- or no CAR (w/o) in triplicates. Transduction efficiency was analyzed 4 d post transduction by quantification of the GFP positive cells by flow cytometry. (**A**) Analysis of the LV batch used for the experiments of Figure 2 and Figure 4. ns, not significant (P= .999 [CD19-CAR/w/o], P= .999 [CD20-CAR/w/o], P= .999 [CD318-CAR/w/o]). (**B**) Analysis of the LV batch used for the experiments of Figure 3 and Figure 5. *P (P= .0476 [CD19-CAR/w/o], P= .0285 [CD318-CAR/w/o]), **P .0057 . Ordinary one-way ANOVA with multiple comparisons.

Figure S9:

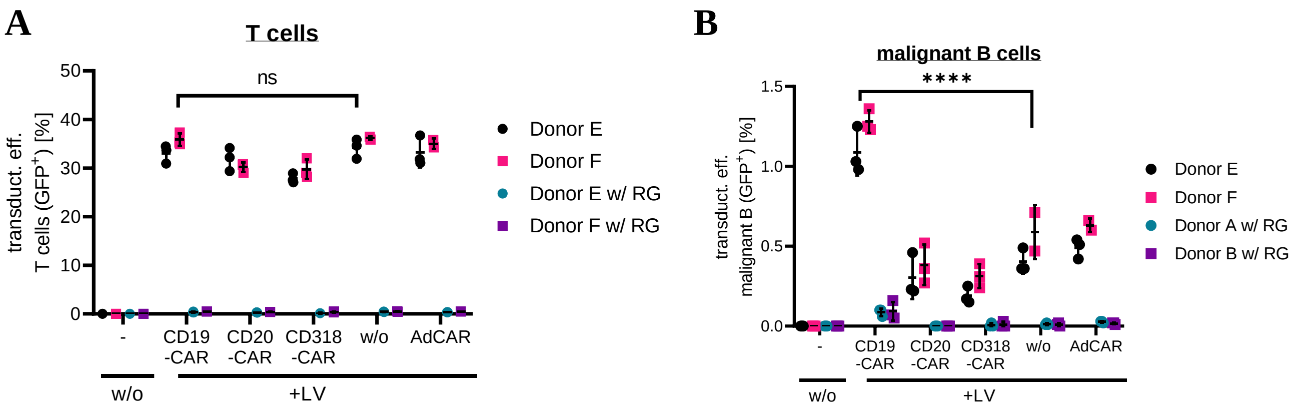

Figure S9. Transduction of malignant B cells in presence of activated T cells.
70% malignant B-precursor cells of one B-ALL patient were mixed to 30% activated T cells of two healthy donors. GFP-encoding LVs displaying different CAR proteins and LNGFR were added for 90 min at 37 °C (MOI 5). Unbound LV was removed by four successive washing steps and the transduction efficiency was analyzed 4 days post transduction by flow cytometry. Triplicates from one experiments ± SD are shown. ****P< .0001, ns= not significant P=.9809, ordinary two-way ANOVA.

Figure S10:

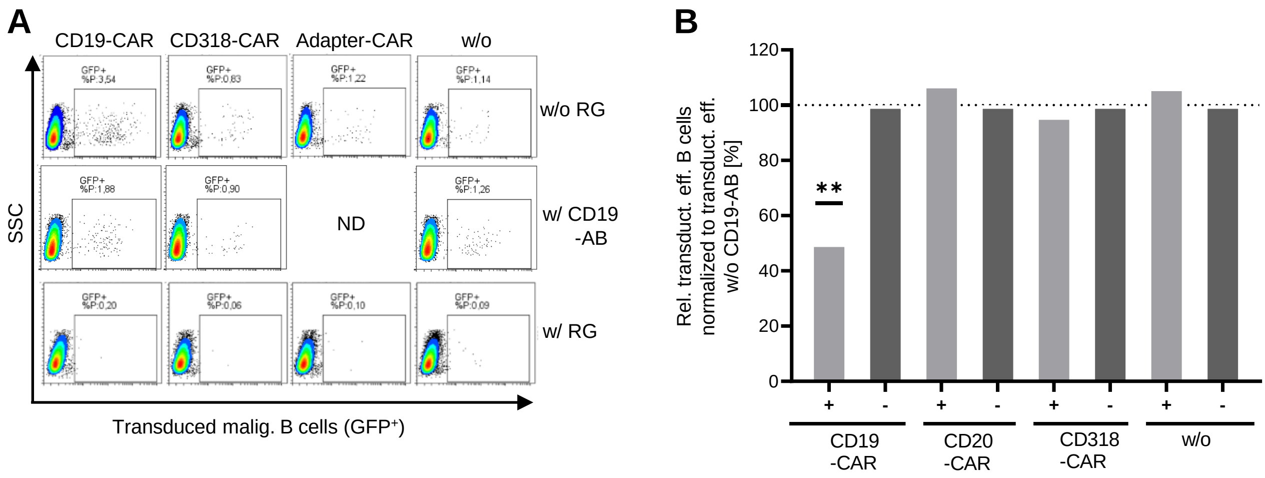

Figure S10. Transduction of malignant B cells with GFP-encoding LVs displaying CARs of different specificities and LNGFR.

30% malignant B-precursor cells of one B-ALL patient were mixed to 70% PBMC of a healthy donor. LV (MOI 40) and the LV-inhibitor Raltegravir were added to respective samples. Additional samples were pretreated with CD19-blocking antibody (1µg/ml) for 30min at 4°C (w/ CD19-AB). GFP-encoding LVs displaying different CAR proteins and LNGFR were added for 90 min at 37 °C. Unbound LV was removed by three successive washing steps and the transduction efficiency was analyzed 4 days post transduction by flow cytometry. (**A**) Transduction of malignant B cells co-cultured with PBMC is shown (representative data of triplicates of one donor). Respective samples were treated with Raltegravir (w/ RG) or left untreated (w/o RG). (**B**) Transduction of B cells in presence or absence of CD19-AB normalized to transduction in absence of antibody. **P= .0024, student t test. ND, not determined

Supplemental Tables

Table S1. Coverage of viral protein sequences by LC-MS/MS in purified GMP-grade LV preparations.

**Viral/CAR protein**		**Protein length (amino acids)**	**CD19-CAR LV**			
			**Lot 1**	**Lot 2**	**Lot 3**	**Lot 4**
**Gag**	MA (matrix protein)	130	76%	70%	67%	76%
	CA (capsid protein 24)	230	79%	76%	46%	88%
	p2	15	ns	ns	ns	ns
	p6	51	100%	100%	100%	100%
**Pol**	PR (Peptidase A2)	69	69%	69%	69%	69%
	RNase H	123	51%	41%	ns	60%
	RT (reverse transcriptase)	190	54%	46%	36%	55%
	IN (integrase)	150	22%	30%	20%	27%
**VSV-G Env**		511	52%	53%	39%	61%
**CD19-scFV**	CD19 light chain	107	22%	29%	22%	31%
	CD19 heavy chain	131	ns	ns	ns	21%
**CAR**	CD8 transmembrane domain	72	14%	14%	14%	14%
	41BB costim	42	ns	ns	ns	14%
	CD3 zeta domain	112	89%	88%	88%	91%

Gag: group-specific antigen, Pol: polymerase, Env: envelope, CAR: chimeric antigen receptor, LV: lentiviral vector
